# Opt1 imports CoA precursors as glutathione mixed disulfides

**DOI:** 10.1016/j.jbc.2025.110503

**Published:** 2025-07-21

**Authors:** Jouke Jan Wedman, Lotte de Vries, Bart van Lingen, Marianne van der Zwaag, Rubén Gómez-Sánchez, Ralph Hardenberg, Wim Huibers, Hjalmar Permentier, Erick Strauss, Michael Chang, Fulvio Reggiori, Anton I. de Kroon, Ody C.M. Sibon, Hein Schepers

**Affiliations:** 1 Department of Biomedical Sciences, University of Groningen, University Medical Center Groningen, Groningen, the Netherlands; 2 Interfaculty Mass Spectrometry Center, Groningen Research Institute of Pharmacy, University of Groningen, Groningen, the Netherlands; 3 Department of Biochemistry, Stellenbosch University, Stellenbosch, South Africa; 4 European Research Institute for the Biology of Ageing, University of Groningen, University Medical Center Groningen, Groningen, the Netherlands; 5 Department of Biomedicine, Aarhus University, Aarhus, Denmark; 6 Molecular Biophysics & Biochemistry, Bijvoet Center for Biomolecular Research and Institute of Biomembranes, Utrecht University, Utrecht, the Netherlands

**Keywords:** coenzyme A, metabolism, pantetheine, screen, yeast

## Abstract

Pantothenate is a key vitamin for the intracellular biosynthesis of the essential molecule coenzyme A (CoA). Pantothenate can be biosynthesized or is taken up by cells *via* plasma membrane transporters. In the cell, pantothenate, ATP, and cysteine are required to synthesize CoA *via* five enzymatic steps. This canonical CoA biosynthesis route is well-studied in various organisms. Alternative routes that begin with the uptake of pantetheine (PanSH) or 4′-phosphopantetheine (PPanSH) as initial CoA precursors also exist. These alternative routes are vital for numerous unicellular organisms and are of interest for treating human diseases caused by defects in the canonical CoA biosynthesis pathway. In contrast to the uptake mechanisms for pantothenate, the cellular uptake mechanisms for PanSH and/or PPanSH are unresolved. Through a combination of *in vivo* experiments, yeast genetics, and the use of chemically traceable compounds, we uncovered a non-canonical CoA biosynthesis pathway. We demonstrate that extracellularly, PanSH and PPanSH form mixed disulfides with glutathione, followed by uptake *via* the oligopeptide transporter Opt1. Once PanSH or PPanSH are imported, they are converted into CoA. Via this route, several proteins essential for the canonical pantothenate-cysteine-dependent CoA biosynthesis pathway become dispensable. Additionally, we show that yeast strains cultured on PanSH or PPanSH have a growth advantage under conditions of decreased cysteine biosynthesis. The identified non-canonical CoA biosynthesis route provides a framework to treat CoA-linked diseases and to manipulate the growth of pathogenic or beneficial organisms that grow on PanSH or PPanSH.

The metabolic cofactor coenzyme A (CoA) is essential to all organisms, ranging from prokaryotes to plants and animals. CoA is required for the citric acid cycle, fatty acid biosynthesis, and fatty acid degradation (1, 2, 3). Post-translational modifications, including acylation, CoAlation, and 4′-phosphopantetheinylation, also depend on CoA (4).

For more than 70 years, the prevailing dogma has been that in most organisms, CoA biosynthesis begins with the biosynthesis or uptake of pantothenate (vitamin B5, Pan) (Fig. 1). Through five consecutive enzymatic steps, pantothenate is converted intracellularly into CoA. First, pantothenate kinase (PANK) phosphorylates pantothenate to form 4′-phosphopantothenate (PPan). Next, phosphopantothenoylcysteine synthetase (PPCS) adds a cysteine moiety, creating 4′-phosphopantothenoylcysteine (PPanCySH). This is then followed by decarboxylation into 4′-phosphopantetheine (PPanSH) by phosphopantothenoylcysteine decarboxylase (PPCDC). Finally, PPanSH is converted into CoA by CoA synthase (COASY), a bifunctional enzyme that carries out the last two steps in CoA biosynthesis: phosphopantetheine adenylyltransferase (PPAT) and dephospho-CoA kinase (DPCK) (2). Since the elucidation of this canonical pathway, several mutations in PANK (5), PPCS (6), PPCDC (7), and COASY (8), causative for lethal neurological or cardiac diseases, have been identified. These findings have renewed interest in the CoA biosynthesis pathway to discover potential therapies to treat these pathologies (9).

**Figure 1:**
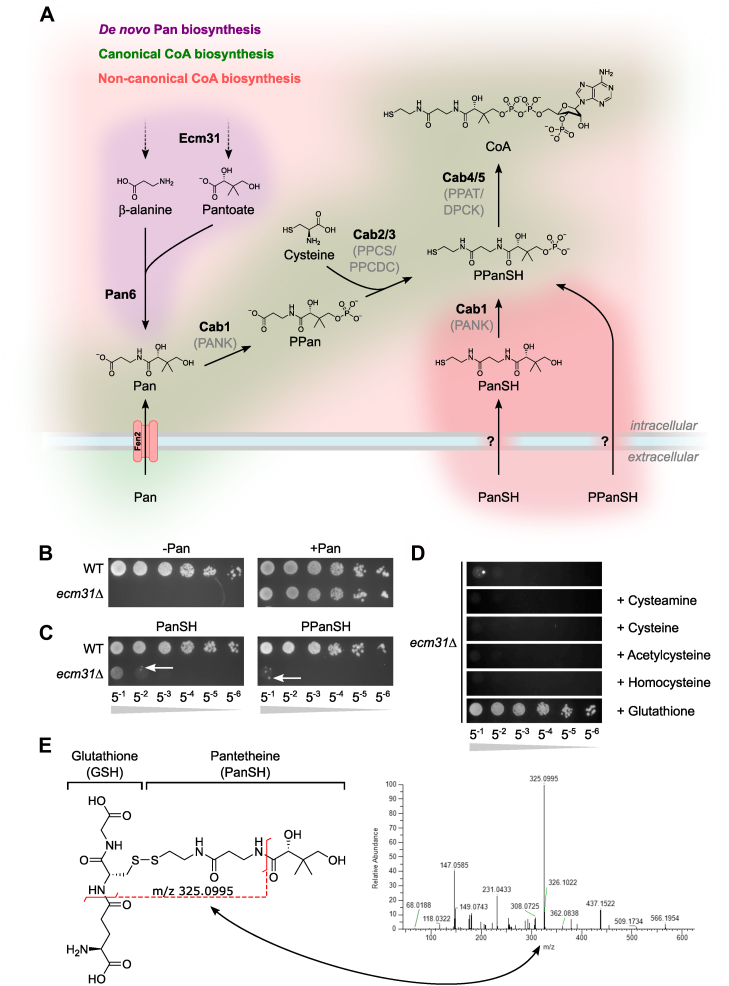
**Co-supplementation of glutathione enables the growth of *ecm31*Δ on (P)PanSH.***A*, cartoon depicting the *de novo* biosynthesis (*purple* background), uptake of pantothenate and its canonical conversion into CoA (*green* background), and non-canonical CoA biosynthesis *via* the uptake of (P)PanSH (*red* background). Fen2 is the identified transporter for pantothenate; transporters for PanSH and PPanSH in *S. cerevisiae* are unknown. Names of enzymatic activity are given in brackets underneath the yeast protein name. Pan; pantothenate, PPan; phosphopantothenate, PanSH; pantetheine PPanSH; 4′-phosphopantetheine, CoA; coenzyme A, Cys; cysteine, PANK; pantothenate kinase, PPCS; phosphopantothenatecysteine synthetase, PPCDC; phosphopantothenoylcysteine decarboxylase, PPAT; phosphopantetheine adenylyltransferase, DPCK; dephospho-CoA kinase. *B*, Spot test of wild-type (Y00000) and *ecm31*Δ (Y03316) yeast strains on pantothenate-free media supplemented without or with pantothenate (27 μM). Strains were grown for 6 days at 30 °C. *C*, spot test of wild-type (Y00000) and *ecm31*Δ (Y03316) yeast strains on pantothenate-free media supplemented with PanSH or PPanSH (both 27 μM). The white arrows highlight the growth of suppressor strains. Strains were grown for 6 days at 30 °C. *D*, chemical screen of the *ecm31*Δ strain (Y03316) in a spot test on pantothenate-free media supplemented with PanSH, co-supplemented with cysteamine, cysteine, acetylcysteine, homocysteine, or glutathione (67 μM). Strains were grown for 4 days at 30 °C. Representative images of n ≥ 3. For all spot tests, the strains were harvested, washed, and corrected to an OD_600_ of 1.00. A serial dilution (depicted by the declining grey slope) was made in 5-fold increments from 5^−1^ to 5^−6^ and 5 μl aliquots of each dilution were spotted. *E*, molecular structure of the mixed disulfide of PanSH and GSH. The LC-MS/MS reveals a unique fragment with an m/z value corresponding to the mixed disulfide of (P)PanSH and GSH ((P)PanSSG).

While knowledge of the pantothenate-dependent CoA biosynthesis pathway is continuously increasing, pantothenate-independent biosynthesis routes remain largely unexplored, although evidence is accumulating proving their existence. In 1951, it was demonstrated that bacteria from the genus *Lactobacillus* require pantethine (PanSSPan, the disulfide of pantetheine (PanSH)) for growth instead of pantothenate (Fig. 1) (10, 11). Type I/II PANKs are promiscuous enzymes because, in addition to phosphorylating pantothenate into PPan, they also phosphorylate PanSH into 4′-phosphopantetheine (PPanSH) (Fig. 1) (12). Recently, we described that certain microbiome species are more efficiently converting PanSH to PPanSH than the canonical pathway converts pantothenate to PPanSH (13). The biological relevance of this alternative pathway to CoA is further underscored by evidence that cells and animals (including models of CoA-linked diseases) with defects in PANK, PPCS, or PPCDC can survive on, benefit from, or depend on PanSH (14, 15) or PPanSH (13, 16). Therefore, mechanisms must exist that enable the passage of PanSH and PPanSH over biological membranes into cells, and the aim of this study was to elucidate these mechanisms.

To identify potential uptake mechanisms for PanSH and PPanSH, we utilized auxotrophic *Saccharomyces cerevisiae* (baker’s yeast) strains that cannot biosynthesize pantothenate *de novo*. The growth of this strain relies on the presence of pantothenate in the medium. This strain did not grow in medium where pantothenate was replaced by PanSH or PPanSH. PanSH forms mixed disulfides *in vitro* with various small thiols, and hypothetically, this could also occur *in vivo* (17). Based on these studies, we hypothesized that while cells may have a limited capacity to take up PanSH or PPanSH (either in its reduced form or as a homodimer), mixed disulfides of PanSH or PPanSH could potentially be taken up more readily. Using the pantothenate auxotrophic yeast strain, we conducted a screen where we co-supplemented PanSH with various small thiols and found that co-supplementation of glutathione (GSH) with either PanSH or PPanSH resulted in a robust rescue of growth. We demonstrate that PanSH and PPanSH can form a mixed disulfide with GSH, and this mixed disulfide is subsequently transported into the cell by the Opt1 transporter. Once taken up, PanSH is converted into CoA in a Cab2-(PPCS), Cab3-(PPCDC) independent manner, while PPanSH uptake also bypasses the need for Cab1 (PANK). Finally, we discovered that yeast cultured on PanSH or PPanSH shows a growth advantage under conditions of impaired biosynthesis of cysteine, a metabolite that is bypassed in this non-canonical CoA biosynthesis route (Fig. 1).

Our data reveal a mechanism for the uptake of the alternative CoA precursors PanSH and PPanSH in a eukaryotic cell, demonstrating the presence of a pantothenate-independent non-canonical CoA biosynthesis pathway important for the maintenance of CoA levels in organisms and thus for life.

## Results

### Co-supplementation of glutathione enables the growth of *ecm31*Δ on (P)PanSH

To test whether *S. cerevisiae* can take up mixed disulfides of PanSH or PPanSH, we took advantage of two auxotrophic *S. cerevisiae* strains, *ecm31*Δ and *pan6*Δ. Ecm31 is required for pantoate biosynthesis, while Pan6 ligates pantoate with β-alanine to form pantothenate. This makes *ecm31*Δ and *pan6*Δ unable to endogenously synthesize pantothenate (Fig. 1, *A* and *B*) (18). Both strains grow on medium supplemented with pantothenate. However, when both strains were cultured on solid synthetic media lacking pantothenate and supplemented with PanSH or PPanSH, no growth was observed, with the exception of suppressor colonies, which appeared after prolonged culture (6–8 days) (Fig. 1*C*, white arrows, Fig. S1). For the remainder of this article, in case PanSH and PPanSH are both applicable, this will be referred to as (P)PanSH.

Since Brown & Snell demonstrated that PanSH can form a mixed disulfide with other thiols (17), we performed a screen in which we co-supplemented PanSH with small thiols (cysteamine, cysteine, acetylcysteine, homocysteine, or glutathione). We observed a full rescue of the *ecm31*Δ strain on PanSH when GSH was co-supplemented, comparable to the growth on pantothenate (Fig. 1*D*). Since it has been described that GSH and PanSH can form PanSSG under oxidizing conditions (17, 19), we argued that this rescue could be the result of the uptake of a mixed disulfide of PanSH and GSH (PanSSG). We confirmed the spontaneous formation of PanSSG by LC-MS/MS experimentally (Figs. 1*E* and S2). Co-supplementation of GSH also enabled growth on PPanSH as well but not dephosphoCoA (dePCoA) or CoA (Fig. S3, yellow boxes).

### Growth of *ecm31*Δ by (P)PanSH and GSH co-supplementation depends on *OPT1*

**Figure 2:**
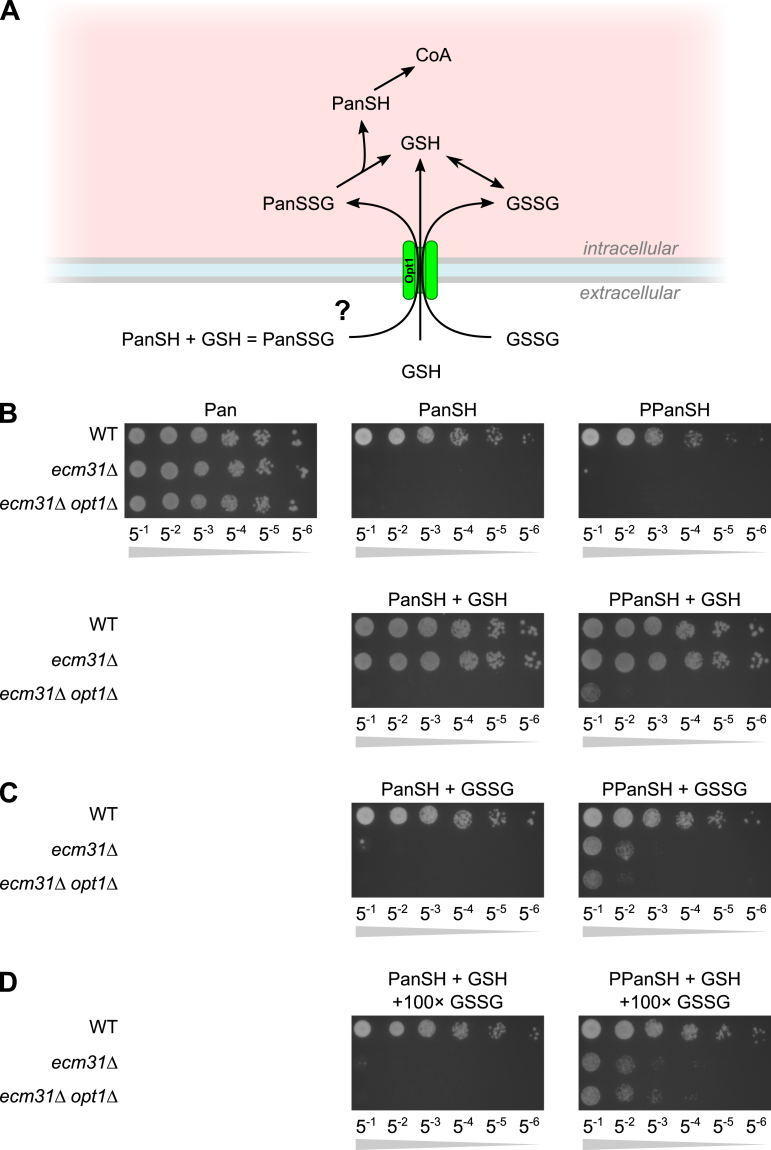
**Growth of *ecm31*Δ by (P)PanSH and GSH co-supplementation depends on *OPT1*.***A*, Cartoon depicting the uptake of GSH, GSSG, and PanSSG by Opt1. *B*, spot test of wildtype (Y00000), *ecm31*Δ (Y03316), and *ecm31*Δ *opt1*Δ (JWY100) yeast strains on pantothenate-free media supplemented with pantothenate, PanSH, or PPanSH (all 27 μM), and co-supplemented with or without GSH (67 μM). *C*, spot test of wildtype (Y00000), *ecm31*Δ (Y03316), and *ecm31*Δ *opt1*Δ (JWY100) yeast strains on pantothenate-free media supplemented with pantothenate, PanSH, or PPanSH (all 27 μM), and co-supplemented with GSSG (33.5 μM). *D*, spot test of wildtype (Y00000), *ecm31*Δ (Y03316) and *ecm31*Δ *opt1*Δ (JWY100) yeast strains on pantothenate-free media supplemented with pantothenate, PanSH, or PPanSH (all 27 μM), co-supplemented with GSH (67 μM) as well as a 100× molar excess of GSSG (3.35 mM). Representative images of n ≥ 3.

Next, we aimed to identify a possible uptake mechanism for (P)PanSSG and we postulated that a transporter would be required for its uptake. We argued that Opt1 is a possible candidate transporter of (P)PanSSG for two reasons: the plasma membrane-localized Opt1 transporter is the sole importer of extracellular glutathione (GSH) and oxidized glutathione (GSSG) in *S*. *cerevisiae* (20), and Opt1 also imports other conjugates of GSH (20, 21) (Fig. 2*A*). To investigate the role of Opt1 in the rescue, we knocked-out *OPT1* in the *ecm31*Δ background. This strain showed no rescue phenotype when GSH was co-supplemented with (P)PanSH (Fig. 2*B*), and this strain regained the rescue phenotype when complemented with a plasmid copy of *OPT1* (Fig. S4). Co-supplementation of GSSG with (P)PanSH did not rescue the *ecm31*Δ strain (Fig. 2*C*). Co-supplementation of an excess of GSSG to (P)PanSH with GSH resulted in impaired growth, suggesting competitive inhibition of the uptake of (P)PanSSG by GSSG by Opt1 (Fig. 2*D*).

### Growth of *ecm31*Δ by (P)PanSH and GSH co-supplementation depends on pre-oxidized medium

**Figure 3:**
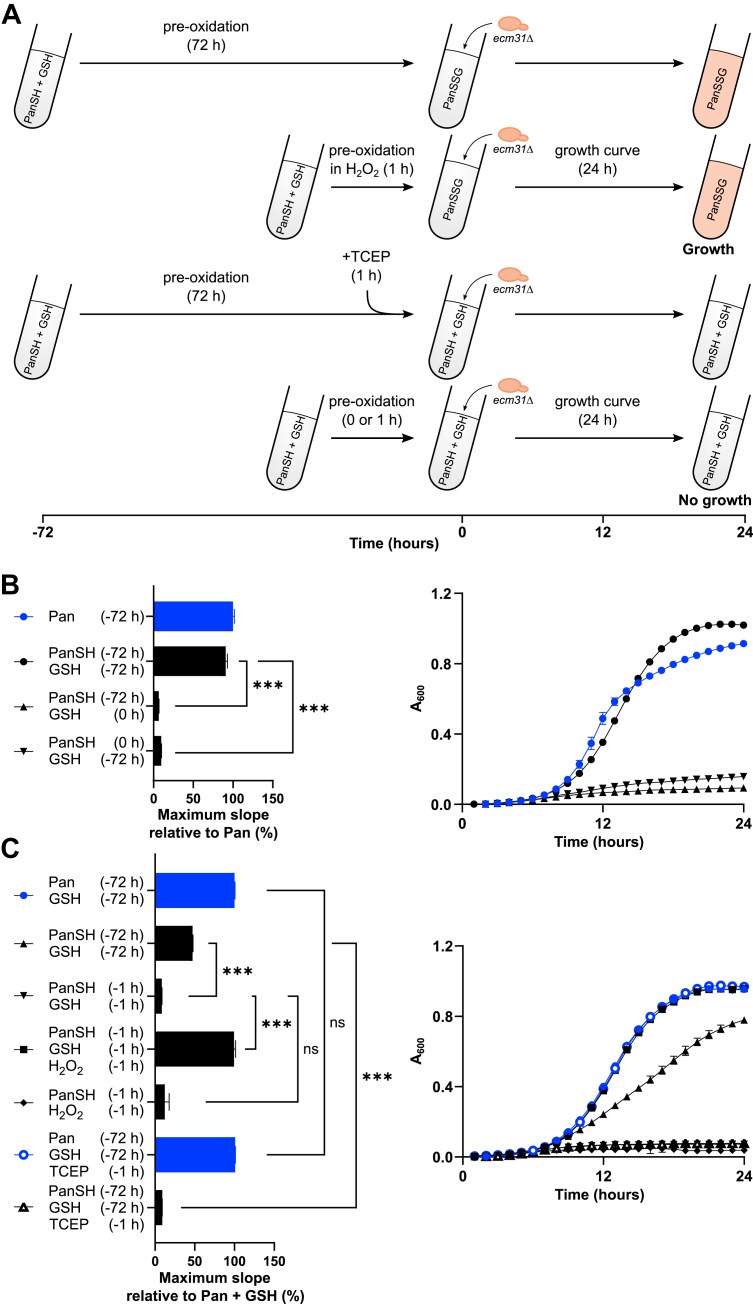
**Growth of *ecm31*Δ by (P)PanSH and GSH co-supplementation depends on pre-oxidized medium.***A*, the upper cartoon depicts the predicted growth of *ecm31*Δ as a result of pre-oxidation of PanSH with GSH for 72 h, or the addition of H_2_O_2_ (40 μM) for 1 h prior to the start of a 24 h growth curve. The lower cartoon depicts the predicted absence of growth of *ecm31*Δ when no pre-oxidation is performed, or when TCEP (100 μM) is added after pre-oxidation 1 h prior to the start of a 24 h growth curve. *B*, *Left* graph shows the maximum slopes of the growth curves of *ecm31*Δ (Y03316) cultured with pantothenate or PanSH (both 27 μM) co-supplemented with GSH (67 μM), relative to pantothenate (set to 100%). *Right* graph shows the corresponding growth curves. Included are controls in which either PanSH or GSH had been pre-oxidized, with GSH and PanSH co-supplemented at the start of the growth curve, respectively. Pre-oxidation was performed directly in the media. *C*, the *left* graph shows the maximum slopes of the growth curves of *ecm31*Δ (Y03316) cultured with pantothenate or PanSH (both 27 μM) co-supplemented with GSH (67 μM), relative to pantothenate (set to 100%). Included are conditions where pre-oxidation was shortened to 1 h and H_2_O_2_ (40 μM) was included, as well as conditions in which TCEP (100 μM) was added 1 h prior to the start of the growth curve in medium pre-oxidized for 72 h. *Right* graph shows corresponding growth curves. Pre-oxidation was performed in water. Data is shown as mean ± SD of three biological replicates. ∗∗∗*p* < 0.0001, unpaired one-tailed *t* test. TCEP, Tris(2-carboxyethyl)phosphine; H_2_O_2__,_ hydrogen peroxide.

We wanted to exclude the possibility that the uptake of the GSH molecule somehow results in the uptake of (P)PanSH, independent of mixed disulfide formation. The use of liquid media enabled us to control the time GSH and (P)PanSH can react and form mixed disulfides by combining these compounds at different time points before the start of a growth curve (pre-oxidation) (Fig. 3*A*). We observed that a 72-h pre-oxidation of PanSH and GSH is required for both the formation of the mixed disulfide (Fig. S5) and the growth of the *ecm31*Δ strain (Fig. 3*B*). Solely pre-oxidizing PanSH and adding GSH later at the start of the growth curve (or *vice versa*) showed no benefit, *i.e*., the benefit of pre-oxidation depends on both PanSH and GSH being present (Fig. 3*B*). Similar results were observed with PPanSH and GSH (Fig. S6, *A* and *B*). Moreover, the addition of TCEP (tris(2-carboxyethyl)phosphine, a reducing agent) 1 h before the start of the growth curve abolished the growth benefit of the 72-h pre-oxidation. This absence of growth was not due to toxicity, because the addition of TCEP did not affect growth on pantothenate (Fig. 3*C*). In contrast, the addition of H_2_O_2_ (hydrogen peroxide, an oxidizer) shortened the required pre-oxidation time of PanSH with GSH to 1 h, while showing no effect in PanSH only (Fig. 3*C*). In these latter experiments, pre-oxidation of PanSH with GSH was done only in water (which was added to the synthetic media at the start of the growth curve), further proving that the required reaction is between PanSH and GSH only (Fig. 3*C*). Similar results were obtained with PPanSH (Fig. S6*C*). As anticipated, the rescue by (P)PanSH and GSH in pre-oxidized medium disappeared in *opt1*Δ cells (Fig. S7), corroborating that Opt1 is a transporter of (P)PanSSG. The dependence on Opt1 furthermore demonstrates that the rescue is not due to the extracellular breakdown of (P)PanSH to pantothenate (which can be taken up by the pantothenate transporter Fen2), somehow facilitated by GSH.

### Co-supplementation of GSH with (P)PanSH bypasses the requirement for canonical CoA biosynthesis proteins

**Figure 4:**
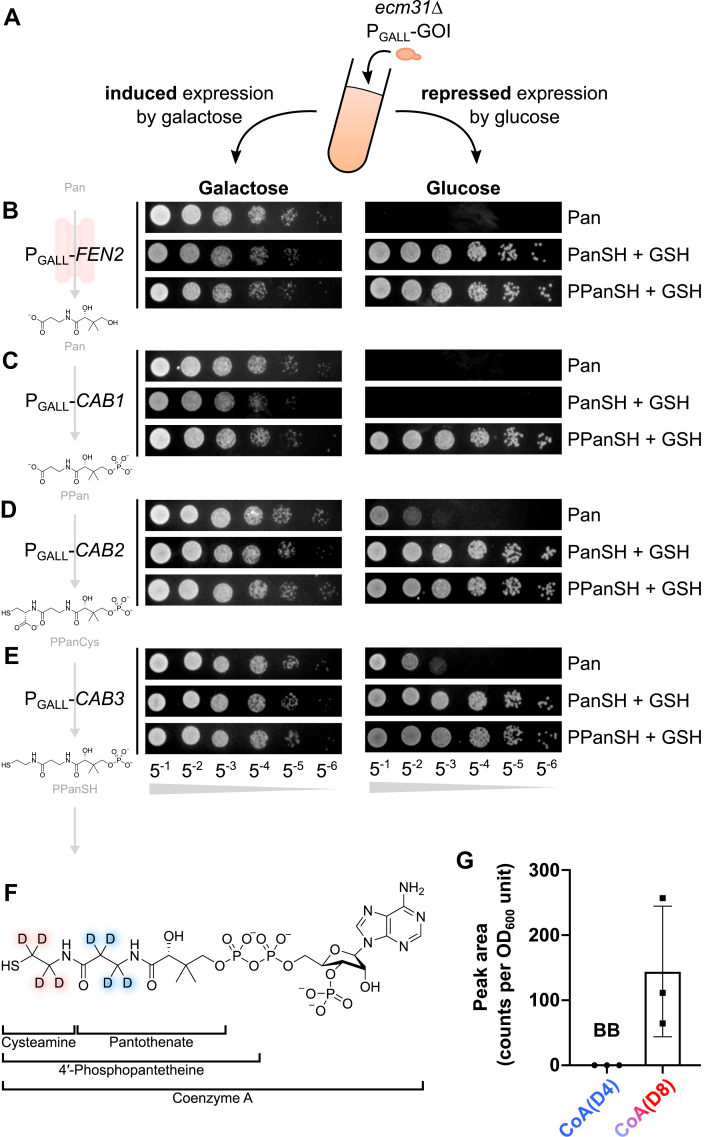
**Co-supplementation of GSH with (P)PanSH bypasses the requirement for canonical CoA biosynthesis proteins.***A*, cartoon depicting the experiments done using P_GALL_ fusion strains. GOI = Gene Of Interest. Spot test of the (*B*) *ecm31*Δ P_GALL_-*FEN2* strain (JWY156), (*C*) *ecm31*Δ P_GALL_-*CAB1* strain (JWY159), (*D*) *ecm31*Δ P_GALL_-*CAB2* strain (JWY162), and (*E*) *ecm31*Δ P_GALL_-*CAB3* strain (JWY165) on pantothenate-free media supplemented with Pan (27 μM), PanSH (27 μM) + GSH (67 μM), or PPanSH (27 μM) + GSH (67 μM). Media contained either galactose or glucose as carbon source. Strains were grown for 2 days at 30 °C. Representative images of n ≥ 3. *F*, structure of CoA(D8). Deuterium labels in the pantothenate moiety are depicted in *blue*, and deuterium labels in the cysteamine moiety are depicted in *red*. When PPanSH(D8) is supplemented and all labels are found in CoA, the cysteamine moiety is retained during its conversion into CoA. *G*, bar chart showing the amount of CoA(D4) *versus* CoA(D8) measured in *ecm31*Δ (Y03316) cultured in pre-oxidized SD media with PPanSH(D8) (27 μM) and GSH (67 μM). Data is shown as mean ± SD of three biological replicates. BB, Below Background.

To provide additional evidence that (P)PanSH is directly taken up by Opt1 and not degraded into PanSH or pantothenate, we created strains deficient of proteins responsible for the uptake of pantothenate (Fen2) and its sequential conversion into PPanSH (Cab1, Cab2, and Cab3) (22, 23). To this end, we genetically swapped the endogenous promoters of the respective genes with the galactose-inducible GALL promoter (24) in the *ecm31*Δ background. These strains were cultured in media with either galactose or glucose as a carbon source for expression or repression of the gene of interest, respectively (Fig. 4*A*). As expected, we observed that repression of the expression of *FEN2*, *CAB1*, *CAB2*, or *CAB3* resulted in an inhibition of the growth of *ecm31*Δ when cultured on pantothenate, while either PanSH or PPanSH co-supplemented with GSH rescued this growth defect (Fig. 4, *B* and *E*). The expected exception was repression of *CAB1* (responsible for the phosphorylation of pantothenate and PanSH), in which only PPanSH co-supplemented with GSH resulted in growth (Fig. 4*C*). Similar results were obtained when using a *cab1* thermosensitive mutant (23), which, when cultured at an elevated temperature, only showed growth when cultured on PPanSH co-supplemented with GSH (Fig. S8). To obtain further evidence that the cysteamine moiety of PPanSH is retained during uptake and conversion into CoA, we used stable isotope labeled PPanSH with four deuterium labels in the pantothenate backbone and four deuterium labels in the cysteamine moiety (PPanSH(D8)) (Fig. 4*F*). Analysis by LC-MS revealed that all eight isotopes were retained in intracellular labeled CoA (CoA(D8)), proving that the cysteamine moiety is retained in PPanSH before conversion into CoA (Fig. 4*G*).

### Characterization of suppressors rescued on (P)PanSH after prolonged culture

**Figure 5:**
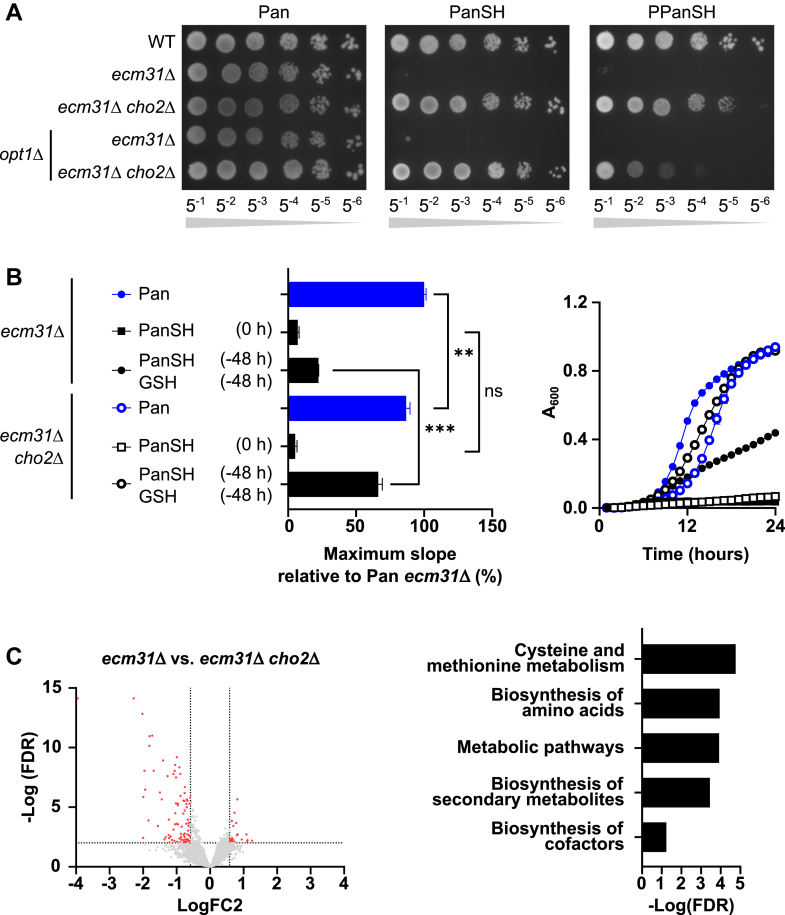
**Characterization of suppressors rescued on (P)PanSH after prolonged culture.***A*, spot test of wildtype (Y00000), *ecm31*Δ (Y03316), *ecm31*Δ *cho2*Δ (JWY025), and the corresponding *opt1*Δ stains (JWY100, JWY101) cultured on pantothenate, PanSH or PPanSH (all 27 μM) for 8 days at 30 °C. Representative images of n ≥ 3. *B*, the *left* graph shows the maximum slopes of the growth curves of *ecm31*Δ (Y03316) and *ecm31*Δ *cho2*Δ (JWY025) cultured on pantothenate or PanSH (both 27 μM), either co-supplemented and pre-oxidized with or without GSH (67 μM), relative to *ecm31*Δ cultured on pantothenate (set to 100%). Pre-oxidation was performed directly in the media. Choline chloride (1 mM) was added to exclude any effects due to alterations of the PE:PC. The *right* graph shows the corresponding growth curves (n = 3). Data are shown as mean ± SD of three biological replicates. ∗∗*p* < 0.005, ∗∗∗*p* < 0.0001, unpaired two-tailed *t* test. *C*, the *left* graph shows a volcano plot depicting differentially expressed genes as determined by RNA-sequencing, comparing *ecm31*Δ (Y03316) with *ecm31*Δ *cho2*Δ (JWY025). The *red* dots depict differentially expressed genes with an FC > 1.5 and an FDR < 0.01. The *right* graph shows the results from the functional GO-analysis (DAVID). Only KEGG-pathways terms with an FDR < 0.05 are shown.

Next, we investigated the characteristics of the suppressors growing on (P)PanSH agar plates after prolonged culture time without co-supplementation of GSH (Fig. 1*C*, white arrows). We collected a library of *ecm31*Δ and *pan6*Δ suppressors that were able to grow on (P)PanSH (Fig. S1) and whole genome sequencing (WGS) revealed that 94% of suppressors in the *ecm31*Δ background shared a 2326delA mutation in *CHO2*, with additional *CHO2* mutations found in both the *ecm31*Δ and *pan6*Δ backgrounds (Table S1, Fig. S9). Cho2 is a methyltransferase that, together with Opi3, methylates phosphatidylethanolamine (PE) into phosphatidylcholine (PC) (25, 26), and its activity is tightly linked to cysteine and sulfur metabolism (27). An engineered *ecm31*Δ *cho2*Δ double mutant also demonstrated growth on (P)PanSH after prolonged culture time (Fig. 5*A*). Additionally, we identified mutants of *MUP1*, which encodes a methionine importer (28), in the suppressor screen (Table S1, Fig. S9). An engineered *ecm31*Δ *mup1*Δ double mutant was also able to grow on (P)PanSH, further validating our screen (Fig. S10*A*). Because of the overrepresentation of *cho2* mutants in the screen, we first focused on this gene and its function. An *OPT1* knockout of the *cho2*Δ suppressor was still able to grow on (P)PanSH, showing that the growth advantage of *cho2*Δ, visible after prolonged culturing, occurs *via* an Opt1-independent manner (Figs. 5*A* and S11). This suggests alternative uptake mechanisms of (P)PanSH that could be related to the membrane lipid composition. We therefore first investigated whether an increased PE to PC ratio (PE:PC) as a result of *cho2* is responsible for the growth advantage on (P)PanSH (Fig. S12*A*). Alterations in the PE:PC have been linked to changes in membrane fluidity and integrity (29, 30), which could explain the increased uptake of certain molecules like (P)PanSH by passive diffusion (31). Lipid composition analysis *via* two-dimensional thin-layer chromatography (2D-TLC) revealed an 8-fold increase in the PE:PC in *cho2*^2326delA^ strains compared to the *ecm31*Δ background (Fig. S12, *B* and *C*). The addition of choline to the medium (enabling PC biosynthesis *via* the Cho2-independent Kennedy pathway (32) (Fig. S12*A*)) restored the PE:PC (Fig. S12, *B* and *C*) but did not prevent the rescue of the *cho2* suppressor on (P)PanSH (Fig. S12*D*). Although the exact uptake mechanism of (P)PanSH in the *cho2* suppressors remains unknown, we can conclude that an increased PE:PC does not explain its growth advantage.

**Figure 6:**
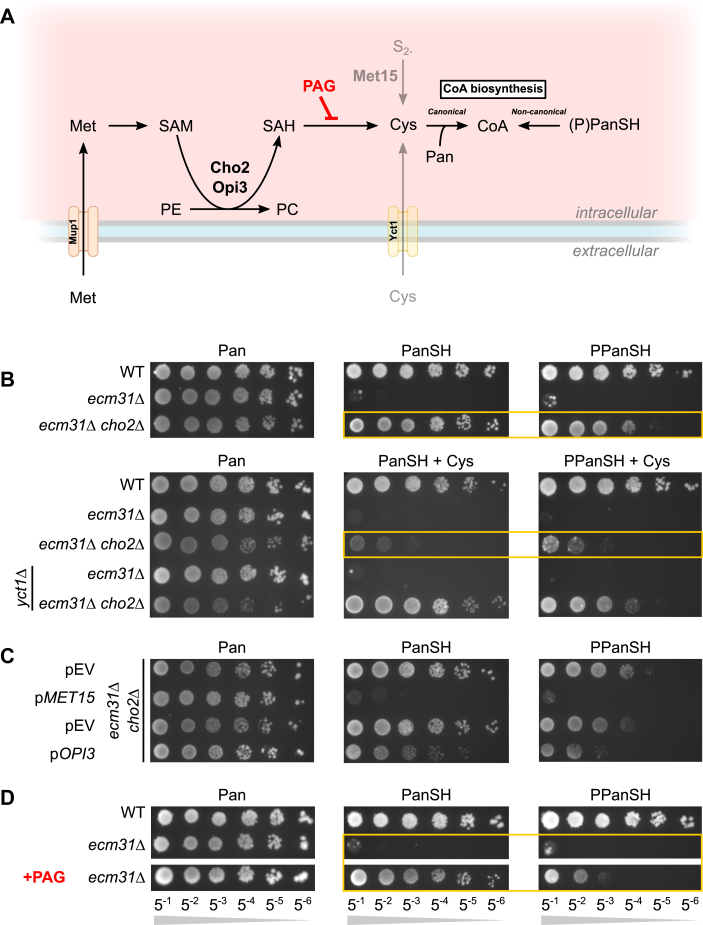
**Yeast cultured on (P)PanSH benefit from reducing cysteine biosynthesis.***A*, cartoon depicting the uptake and *de novo* biosynthesis of cysteine. The inhibitor propargylglycine (PAG) is depicted in *red*. Pathways that apply when cysteine is supplemented or *MET15* is complemented are displayed transparently. *B*, spot test of wildtype (Y00000), *ecm31*Δ (Y03316), *ecm31*Δ *cho2*Δ (JWY025) and corresponding *yct1*Δ strains (JWY088, JWY089) on pantothenate-free media supplemented with Pan, PanSH, or PPanSH (all 27 μM) co-supplemented with or without cysteine (67 μM). Strains were grown for 8 days at 30 °C. The *yellow* boxes depict the reduction in growth caused by cysteine co-supplementation. *C*, spot test of the *ecm31*Δ *cho2*Δ strain (JWY025) carrying either vector that was left empty (pEV), with a copy of *MET15* (p*MET15*), or *OPI3* as multicopy (p*OPI3*). Strains were spotted on pantothenate-free uracil dropout media supplemented with Pan, PanSH or PPanSH (all 27 μM). *D*, spot test of wild-type (Y00000), *ecm31*Δ (Y03316) on pantothenate-free media supplemented with Pan, PanSH, or PPanSH (all 27 μM), and with or without the co-supplementation of propargylglycine (PAG) (320 μM). Strains were grown for 8 days at 30 °C. Choline chloride (1 mM) was added to all plates to exclude any effects due to alterations of the PE:PC. Representative images of n ≥ 3.

In contrast to the growth advantage occurring after prolonged culture on solid medium, growth of the *ecm31*Δ *cho2*Δ suppressor on PanSH was not observed in liquid culture. Interestingly, *ecm31*Δ *cho2*Δ only showed improved growth in liquid culture compared to the *ecm31*Δ background when uptake of PanSH was facilitated *via* GSH co-supplementation (Fig. 5*B*). This suggests that the growth advantage of *cho2*Δ functions *after* PanSH is taken up. To further characterize the effects of the suppressor mutation, we performed transcriptome analysis by RNA-sequencing (GEO accession number GSE290427) and subsequent gene ontology enrichment analysis on differentially expressed genes. In comparison to the *ecm31*Δ background, deletion of *CHO2* in this background resulted in significant alterations in processes related to cysteine and methionine metabolism (Fig. 5*C*, Table S2)*,* in line with previous reports (33, 34). Of note, Cho2 is involved in the biosynthesis of cysteine (27, 33), of which the requirement for canonical CoA biosynthesis is bypassed when (P)PanSH is taken up (Figs. 1 and 6*A*). Considering our observation that other mutations in the suppressor strains can be linked to sulfur (thus cysteine) metabolism either directly (*e.g. met30*, *mup1,* and *sff1*) or indirectly (*e.g. cdc14*, *hrk1*, *ylr012c,* and *gup2*) (Table S1, Fig. S9), we hypothesized that the enhanced growth of *cho2*Δ suppressors is related to altered cysteine homeostasis.

### Yeast cultured on (P)PanSH benefits from reducing cysteine biosynthesis

We next wanted to provide evidence that altered cysteine homeostasis is associated with the growth advantage of the *cho2*Δ suppressor. Phospholipid methylation by Cho2 and Opi3 is a major consumer of S-adenosyl methionine (SAM), yielding S-adenosyl homocysteine (SAH), which can be converted into cysteine (27) (Fig. 6*A*). Importantly, the background strain used in this study is *met15*Δ (Met15 enables cysteine biosynthesis from inorganic sulfide (35)) and is also cultured with methionine, but not cysteine supplementation. Therefore, this yeast strain relies on methylation for cysteine biosynthesis (Fig. 6*A*). As a result, *cho2*Δ strains have decreased cysteine biosynthesis (Fig. S13), as also previously reported (27, 33). Cysteine co-supplementation reduced the growth advantage of the *cho2*Δ suppressor on (P)PanSH, in line with our hypothesis (Fig. 6*B*). Knocking out the main cysteine transporter *YCT1* (36) restored the rescue despite cysteine co-supplementation (Fig. 6*B*, yellow boxes, Fig. S14), strongly suggesting that the ability to grow on (P)PanSH is linked to an intracellular decrease in cysteine biosynthesis. Both overexpressing *OPI3* (which compensates for the methylation deficiency of *cho2*Δ (37) (Fig. S12*C*)) and complementing *MET15,* inhibited the rescue of *cho2*Δ on (P)PanSH (Fig. 6*C*). This is consistent with our hypothesis because these mutations promote *de novo* cysteine biosynthesis (Fig. 6*A*). Moreover, inhibiting cysteine biosynthesis from SAH using propargylglycine (PAG) (38) enabled growth of the *ecm31*Δ background strain on (P)PanSH (Fig. 6*D*, yellow boxes). In line with our hypothesis, these results demonstrate that the growth advantage of *cho2* suppressors when cultured on (P)PanSH is due to impaired cysteine biosynthesis.

## Discussion

The CoA precursor PanSH was discovered in 1933 as the *Lactobacillus bulgaricus* factor (LBF) and was synthesized for the first time in 1950 (10, 39). Since the observation that PanSH is essential for the growth of *L. bulgaricus* (10, 39), evidence that CoA precursors downstream of pantothenate can serve as a starting molecule for CoA biosynthesis has been accumulating in human cells, *Drosophila, C. elegans,* and mice (10, 13, 14, 16, 31). The use of (P)PanSH instead of pantothenate is consistent with the observation that approximately 5% of all microbiome species appear to be missing essential enzymes of the canonical pantothenate-dependent biosynthesis pathway (Fig. S15). Nevertheless, the question of how organisms take up (P)PanSH—considering their polar nature—remained unanswered.

**Figure 7:**
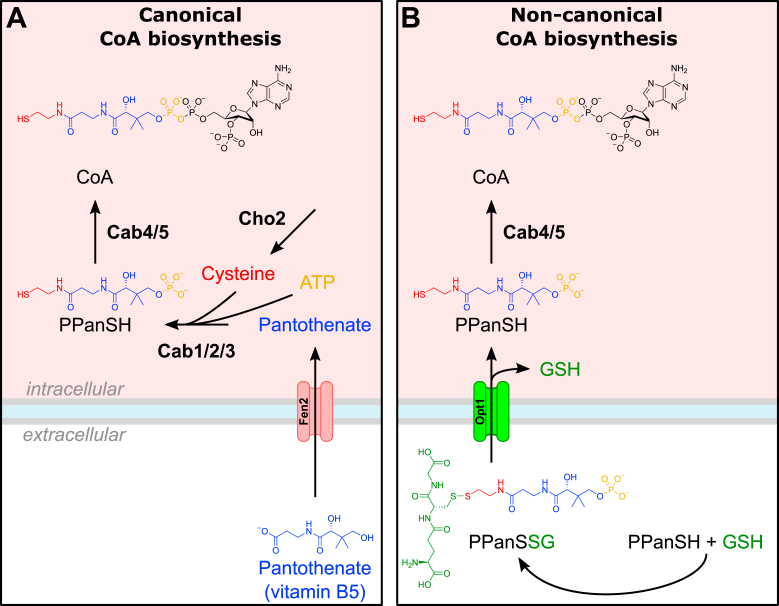
**Graphical summary.***A*, canonical CoA biosynthesis requires uptake of pantothenate *via* Fen2, as well as ATP and cysteine, of which the latter is mainly biosynthesized *via* Cho2. *B*, non-canonical CoA biosynthesis *via* the uptake of glutathione disulfide of (P)PanSH *via* Opt1, bypassing the first enzymes required for CoA biosynthesis, and bypassing the requirement for cysteine biosynthesis *via* Cho2.

In yeast, *de novo* biosynthesis of pantothenate or its uptake *via* Fen2 for canonical CoA biosynthesis has been known for over 2 decades (18, 22) (Fig. 7*A*). Here, we provide evidence that the oligopeptide transporter Opt1 is a transporter for extracellular (P)PanSH as mixed disulfides with GSH in yeast, enabling non-canonical CoA biosynthesis (Fig. 7*B*). We also discovered that yeast that depend on (P)PanSH uptake for CoA biosynthesis have a growth advantage when cysteine biosynthesis is impaired.

During the writing of this manuscript, it was reported that the bacterium *Bacillus subtilis* imports cysteinopantetheine, a mixed disulfide of PanSH and cysteine, *via* the cystine transporter complex TcyJKLMN (40). PPanSH was not investigated in this study in *B. subtilis,* and uptake was observed only when the cystine transporter was overexpressed (40). In our study in *S. cerevisiae*, endogenous levels of Opt1 are sufficient, and we find no evidence supporting the utilization of cysteinopantetheine, since adding cysteine to PanSH does not support the growth of *ecm31*Δ (Fig. 1*D*). Despite the differences between transporters and thiol-containing co-supplements in *S. cerevisiae* and *B. subtilis*, combined, these results independently confirm the ability of cells to uptake PanSH (and in our study PPanSH as well) as mixed disulfides. It is tempting to speculate that uptake of other combinations of (P)PanSH with thiol-containing molecules exists in other organisms as well.

The following question remains: Why do yeast cells that use (P)PanSH as a CoA source have a growth advantage when cysteine biosynthesis is impaired (Figs. 5 and 6)? Interestingly, recently published work shows that impaired CoA biosynthesis leads to elevated cysteine levels, suggesting that canonical CoA biosynthesis is a major cysteine consumer (41). As such, when CoA biosynthesis becomes cysteine-independent after (P)PanSH uptake (Figs. 1*A* and 7, *A* and *B*), cysteine will also likely accumulate, which is known to inhibit cell growth (42, 43). Thus, we argue that mutants with reduced cysteine biosynthesis accumulate less cysteine and therefore have a growth advantage on (P)PanSH and emerge as suppressors (Fig. S1). Supporting this hypothesis is the observation that knocking out *MUP1* in the *ecm31*Δ background strain enables growth on (P)PanSH, while showing a strong growth defect on pantothenate (Fig. S10*A*). This is because the growth defect of *mup1*Δ on pantothenate is likely due to sulfur deficiency, considering methionine is the sole sulfur source provided, which is mainly imported by Mup1 (as described previously (28)). In turn, this explains the rescue on (P)PanSH, since cysteine-independent CoA biosynthesis likely compensates for the sulfur shortage (Fig. S10*B*). How then are suppressors, grown long term on agar plates, able to take up (P)PanSH without GSH co-supplementation (Figs. 5*A* and S10*A*)? We hypothesize that the extended culture time (6–8 days) allows for sufficient passive diffusion of (P)PanSH, as described previously (31). This also explains why the benefit of *cho2*Δ was not observed in the liquid culture of just 24 h (Fig. 5*B*).

The observation that decreased cysteine biosynthesis is favorable for growth *via* cysteine-independent CoA biosynthesis underscores the overlooked role cysteine has in CoA biosynthesis — a view shared by others (41, 44). Further studies are needed to further clarify the bidirectional influence of CoA biosynthesis and cysteine homeostasis.

Several decades after the first observations that CoA precursors other than pantothenate could benefit the growth of living organisms, our findings, alongside those of others (40) now explain how these precursors can be taken up from the extracellular environment. Mixed disulfides of (P)PanSH can be taken up by dedicated transporters to support the growth of cells incapable of obtaining CoA *via* pantothenate. This uptake of (P)PanSH as a mixed disulfide could prove useful in the development of therapeutic strategies to treat CoA-linked diseases (PKAN, PPCS-deficiency and PPCDC-deficiency) that require the bypass of the CoA biosynthesis enzymes PANK2 (5), PPCS (6) or PPCDC (7), respectively. Recently, treatment of a patient with a mutation in PPCS with PanSH showed robust restoration of cardiac function (15), and treatment with PPanSH in a PKAN mouse model restored the expression levels of several biomarkers in brain cells from the globus pallidus, as well as improved mitochondrial function (16). Both studies suggest that PanSH and PPanSH enter mammalian cells and restore CoA biosynthesis. This uptake of (P)PanSH could potentially have been mediated *via* a mixed disulfide, although this remains to be investigated. The Opt1 transporter does not appear to have orthologs outside of yeast. However, other GSH transporters have been described as capable of transporting GSH-conjugates, suggesting that this phenomenon may not be limited to the *Saccharomyces* clade (45). The uptake of cysteinopantetheine, a mixed disulfide between cysteine and PanSH, in *B. subtilis* strengthens this notion. Future research will shed light on the evolutionary conservation and practicality of this newly discovered mechanism.

## Experimental procedures

### Reagents and Tools Table

**Table:** 

Reagent or Resource	Source	Identifier
Yeast strains (all BY4741 background, unless indicated)		
Wildtype (WT) BY4741 (*MAT***a***his3*Δ1 *leu2*Δ0 *met15*Δ0 *ura3*Δ0)	EUROSCARF (46)	Y00000
*ecm31*Δ::*KanMX4*	EUROSCARF (46)	Y03316
*pan6*Δ::*KanMX4*	EUROSCARF (46)	Y02304
*ecm31*Δ and *pan6*Δ suppressor strains	This study	See Table S1
*ecm31*Δ::*KanMX4cho2*Δ::0	This study	JWY025
*ecm31*Δ::*KanMX4cho2*^*2326delA*^	This study	JWY011
*ecm31*Δ::*KanMX4mup1*Δ::*NatMX6*	This study	JWY082
*ecm31*Δ::*KanMX4opt1*Δ::*HphNT1*	This study	JWY100
*ecm31*Δ::*KanMX4cho2*Δ::0 *opt1*Δ::*HphNT1*	This study	JWY101
*ecm31*Δ::*KanMX4yct1*Δ::*HphNT1*	This study	JWY088
*ecm31*Δ::*KanMX4cho2*Δ::0 *yct1*Δ::*HphNT1*	This study	JWY089
*ecm31*Δ::*KanMX4* P_GALL_-*FEN2*::*HphNT1*	This study	JWY156
*ecm31*Δ::*KanMX4* P_GALL_-*CAB1*::*HphNT1*	This study	JWY159
*ecm31*Δ::*KanMX4* P_GALL_-*CAB2*::*HphNT1*	This study	JWY162
*ecm31*Δ::*KanMX4* P_GALL_-*CAB3*::*HphNT1*	This study	JWY165
*MAT***a***ura3 his3 cab1*^*G351S*^*(cab1ts)* (CEN.PK background)	(23)	JS91.14-24
Plasmids		
pFA6a-NatMX6	EUROSCARF (24)	P30437
pFA6a-hphNT1	EUROSCARF (24)	P30347
pGSHU	Addgene (47)	72244
pRS416	ATCC (48)	87521
*MET15* in pRS416	This study	
pRS426	ATCC (49)	77107
*OPI3* in pRS426	This study	
pYM-N27	EUROSCARF (24)	P30281
*hphNT1* in pYM-N27	This study	
*CHO2* in pRS416	This study	
*MUP1* in pRS416	This study	
*YCT1* in pRS416	This study	
*OPT1* in pRS416	This study	
Oligonucleotides		
See Table S4		
Chemicals		
DL-Propargylglycine	Sigma-Aldrich	P7888
D-Calcium pantothenate	Thermo Scientific	A0441346
(*R*)-4′-phosphopantetheine (PPanSH)	Syncom	
(*R*)-4′-phosphopantetheine-D8 (PPanSH(D8))	Syncom	
(*R*)-Pantetheine (PanSH)	Sigma-Aldrich	16702
D-Pantethine (PanSSPan)	Sigma-Aldrich	P2125
3′-Dephosphocoenzyme A	Sigma-Aldrich	D3385
Coenzyme A	Sigma-Aldrich	C4780
L-Glutathione reduced (GSH)	Sigma-Aldrich	G4251
L-Glutathione oxidized (GSSG)	Sigma-Aldrich	G4376
L-Methionine	Sigma	M-2893
L-Cysteine	Sigma	C7477
Cysteamine	Sigma-Aldrich	30070
L-Homocysteine	Sigma-Aldrich	69453
*N*-Acetyl-L-cysteine	Sigma-Aldrich	A9165
Choline chloride	Sigma-Aldrich	C7527
5-fluoroorotic acid (5-FOA)	Thermo Scientific	R0812
RNase-Free DNase Set	Qiagen	79254
RNeasy Mini Kit	Qiagen	74104
D(+) - Raffinose pentahydrate	Formedium	RAF02
D(+) - Galactose	Sigma-Aldrich	G0625
D(+) - Glucose anhydrous	Formedium	GLU03
Tris(2-carboxyethyl)phosphine hydrochloride	Merck	C4706
Hydrogen peroxide 30%	Supelco	1.07210
Synthetic defined (SD) media	This study	See Table S3
Ammonium sulfate	Sigma	A4418
Acetic acid (glacial)	Merck	1000631000
7-fluoro-2,1,3-benzoxadiazole-4-sulfonate ammonium salt	Cayman Chemical Company	34564
Software and Algorithms		
Prism 8	Graphpad	https://www.graphpad.com/
Bio-Rad CFX Maestro	Bio-Rad	https://www.bio-rad.com/product/cfx-maestro-software-for-cfx-real-time-pcr-instruments/
Skyline (64-bit) version 24.1.0.199	Skyline	https://skyline.ms/project/home/begin.view

### Analysis of bacterial CoA biosynthesis genes

Uniprot (https://www.uniprot.org) was used to download bacterial genera (Filter: Bacteria (eubacteria) [2]) containing Type I and Type II Pank, coaA, and coaW, respectively. Bacterial genera containing Type III Pank (coaX) were excluded, as this Type III Pank cannot phosphorylate PanSH. Simultaneously, bacterial genera (Filter: Bacteria (eubacteria) [2]) containing bacterial PPCS, PPCDC or combined PPCS-PPCDC (CoaB, coaC, CoaBC, respectively) were downloaded. Lastly, bacterial genera (Filter: Bacteria (eubacteria) [2]) containing bacterial PPAT and DPCK (coaD and CoaE, respectively) were downloaded. All three lists were de-duplicated, and only one species per genus was kept. This left 900 species containing a CoaA CoaW gene, 3073 species containing CoaBC genes, and 3232 species containing CoaDE genes. All three lists were compared for overlap by making a Venn diagram using the following website: http://bioinformatics.psb.ugent.be/webtools/Venn/.

### Strains and media

Yeast cells were either grown in rich media (YP; 1% yeast extract, 2% peptone) with 2% glucose or galactose, or synthetic media (SM) with either 2% glucose (SMD), galactose (SMG), or raffinose (SMR). The composition of the SM is provided (Table S3), in which methionine is always supplied. Pantothenate and all its derivatives were supplemented at a concentration of 27 μM; methionine, cysteine, and glutathione of 67 μM; oxidized glutathione of 33.5 μM; propargylglycine of 320 μM; choline chloride of 1 mM.

### Suppressor selection and genomic DNA isolation

*ecm31*Δ and *pan6*Δ cells were pre-cultured to mid-log phase (OD_600_ ∼0.70–1.0) from an overnight culture in SD media, harvested by centrifugation (6000×*g*, 2 min), washed twice with sterile water, and normalized to an OD_600_ of 1.00. To generate suppressors, 150 μl of the washed strains were plated on agar plates containing PPanSH. Plates were incubated at 30 °C for 6 days. Subsequently, from each strain, 48 suppressors were transferred to a new agar plate containing PPanSH for a secondary selection of an additional 4 days. After this secondary selection, 33 suppressors of each strain were transferred to YPD medium, along with 3 colonies of each parental strain. After this selection procedure, genomic DNA was isolated as described (50) from 32 *ecm31*Δ and 26 *pan6*Δ suppressor strains. The concentration of gDNA was determined by measuring absorbance using an Implen NanoPhotometer.

### Whole genome sequencing

A pooled sequencing library with a mean insert size of 539 bp was constructed from 250 ng of genomic DNA (each) using the Illumina Nextera DNA Flex Library prep kit and sequenced with paired-end (2 × 150 bp) runs using an Illumina NextSeq instrument and a 300-cycle Mid Output kit. Final library concentrations were measured using Qubit 3 (Thermo Fisher Scientific) and Agilent 2100 Bioanalyzer (Agilent) equipment. The sequencing reads were aligned against the *S. cerevisiae* reference genome (S288C version R64-1-1). Only suppressors with a minimum mean coverage of 20× were further analyzed (32 *ecm31*Δ and 11 *pan6*Δ suppressor strains). Single-nucleotide variants (SNVs), insertions, and deletions (InDels) were called using GATK v3.8 HaplotypeCaller. Variants present in the parental *ecm31*Δ and *pan6*Δ strains were filtered out from all samples. SNVs were assigned a“FILTER” flag using GATK v3.8 VariantFiltration using the following settings "--filterName SNP_LowQualityDepth --filterExpression ‘QD<2.0’ --filterName SNP_MappingQuality --filterExpression ‘MQ<40.0’--filterName SNP_StrandBias --filterExpression ‘FS>60.0’ --filterNameSNP_HaplotypeScoreHigh --filterExpression ‘HaplotypeScore>13.0’--filterName SNP_MQRankSumLow --filterExpression ‘MQRankSum<−12.5’ --filterName SNP_ReadPosRankSumLow --filterExpression‘ReadPosRankSum<−8.0’ --clusterSize 3 -clusterWindowSize 35". For further analysis, only SNVs with the “FILTER” flag set to “PASS” were considered.

### Strain construction

*CHO2* was knocked out or mutated to *cho*^*22326delA*^ in *ecm31*Δ cells by the pCORE strategy, as previously described (47). Briefly, pGSHU containing the URA3 selection marker was amplified by PCR, and its product was transformed into *CHO2*. In a subsequent transformation, the cassette was removed by hybridized oligonucleotides, which either removed the gene or introduced the mutation, and counter-selection was performed on 5-Fluoroorotic acid (5-FOA; 4.2 mM). Other knockouts were introduced using standard procedures using the natMX or hphNT1 antibiotic marker. Swapping the promoter of genes of interest by pGALL was performed using the pYM-N27 plasmid (24), of which the natMX marker was swapped for hphNT1. Transformants were checked for correct integration by colony PCR. Oligonucleotides used are provided (Table S4).

### Growth assays

Cells were pre-cultured to mid-log phase (OD_600_ between 0.70 and 1.00) from an overnight culture in SD, harvested by centrifugation (6000×*g*, 2 min), washed twice with sterile water, and normalized to an OD_600_ of 1.00. In the case of strains with a pGALL induction system, SMR was used to pre-culture the strains. For spot tests, a serial dilution was made in 5-fold increments from 5^−1^ to 5^−6^, and 5 μl aliquots of each dilution were spotted onto agar plates containing the indicated media. Plates were incubated at 30 °C. All comparisons made are within the same experiment on the same day. All phenotypes shown are observed at least three times (n ≥ 3), and representative images are shown. For growth curves, a Costar 48-well plate was used with 500 μl medium, starting with an OD_600_ of 0.02. A Biotek Synergy HTX plate reader was used to measure the absorbance at 600 nm every 20 min for 36 h at 30 °C. Slopes of the linear regression line were calculated, and a student’s *t* test was used to statistically compare the maximum slope values between growth curves.

### Lipid extraction and separation of phospholipids by 2D-TLC

Lipids were extracted as described previously from 20.00 OD_600_ units of yeast grown to OD_600_ 1.00 (51). Extracted lipids were dissolved in 100 μl chloroform: methanol (2:1). Next, 15 μl of the lipid extract was applied on silica gel plates (Merck 1.05641), freshly impregnated with 2.4% (w/v) boric acid. The eluent for the first dimension contained chloroform: methanol: 25% ammonia (71:30:4, v/v/v). After drying under a flow of nitrogen for 30 min, the plate was run in the second dimension using chloroform: methanol: acetic acid (70:25:10, v/v/v) as the eluent. The lipid spots were visualized by iodine staining. Spots were scraped off, and phospholipid classes were quantified as described previously (52). A Student’s *t* test was used to statistically compare these ratios.

### HPLC-MS measurements of isotope labeled CoA

SD supplemented with PPanSH(D8) and GSH was pre-oxidized for 72 h at 30 °C before culture. *ecm31*Δ (Y03316) was cultured to mid-log phase (OD_600_ between 0.70 and 1.0) and 10 OD_600_ units were harvested by centrifugation (6000×*g*, 2 min) and washed twice with water. The cell pellet was resuspended in 100 μl of ice-cold PBS. Cells were sonicated for 20 s at 50 W with a Branson Digital Sonifier SFX150 and spun down at 20,000×*g* for 20 min at 4 °C. 50 μl of 50 mM tris(2-carboxyethyl)phosphine (TCEP) was added to 50 μl of the supernatant. After 15 min of incubation at room temperature, 100 μl of saturated ammonium sulfate was added. The sample was spun down at 20,000×*g* for 20 min at 4 °C, after which the supernatant was transferred to a new tube with 15 μl of 12,5% ammonia. A Shimadzu Nexera X2 HPLC system with LC20ADXR binary pumps was interfaced to an AB Sciex API4000 triple quadrupole mass spectrometer. The analysis was performed by liquid chromatography-tandem mass spectrometry (LC–MS/MS). Analyst software version 1.6.2 by AB Sciex was applied in the data processing. HPLC–MS analysis. An HSS-T3 (100 × 2.1 mm with 1.8 μM particles; Waters) reversed-phase column was used to separate the analytes. The column and autosampler temperatures were set at 50 °C and 15 °C, respectively. The injection volume was 5 μl, and the flow was set at 0.38 ml/min. Mobile phase A consisted of water with 20 mM ammonium acetate, mobile phase B consisted of methanol/ultra-pure water (90/10) with 5 mM ammonium acetate. A gradient was used to separate the analytes in 10 min. Mass spectrometric detection (MS ESI) is performed in multiple reaction monitoring (MRM) mode. Acquisition settings in MRM mode were ion spray voltage 4.5 kV; CAD 6; curtain gas 20, gas 1, 40; gas 2, 50, and source temperature, 600 °C.

### HPLC measurements of cysteine

Strains were cultured in synthetic media with pantothenate to mid-log phase (OD_600_ between 0.70 and 1.0). 15 OD_600_ units were harvested by centrifugation (13,000×*g*, 30 s) and the pellet was weighed. HPLC was done as previously described (31), with small adjustments. Pellets were dissolved in 1000 μl water, of which 100 μl was taken and sonicated for 20 s at 50 W with a Branson Digital Sonifier SFX150. Samples were centrifuged for 15 min at 20,000×*g* at 4 °C to collect supernatant. 10 μl Tris(2-carboxyethyl)phosphine hydrochloride (Sigma) 50 mM was added to 50 μl sample supernatant and incubated at RT for 15 min after vortex-mixing. 40 μl of saturated ammonium sulfate solution was added to remove proteins. The samples were centrifuged at 20,000×*g* for 15 min at 4 °C. The clear supernatant 50 μl was derivatized with 45 μl of 7-fluoro-2,1,3-benzoxadiazole-4-sulfonate ammonium salt (SBD-F, Sigma) (1 mg/ml in borax buffer, 0.1 M containing 1 mM EDTA disodium, pH 9.5) and 5 μl of ammonia solution (12.5% vol/vol, Merck Millipore) at 60 °C for 1 h. The derivatized samples were placed in a refrigerated autosampler (10 °C) in the Shimadzu HPLC system and injected for cysteine analysis. Chromatographic analysis was performed with a Shimadzu LC-10AC liquid chromatograph, SCL-10A system controller, SIL-10AC automatic sample injector and LC-10AT solvent-delivery system. A Shimadzu RF-20Axs fluorescence detector was used for derivatized sample extract analysis. The fluorescence detector was set at excitation and emission wavelengths of 385 nm and 515 nm, respectively. Signal output was collected digitally with Shimadzu Labsolution software, and post-run analyses were performed. Chromatographic separation of the analytes was achieved with a Phenomenex Gemini C18 guard column (4 × 3 mm) connected to a Phenomenex Gemini NX-C18 analytical column (4.6 × 150 mm; 3-μm particles) at 45 °C. The two mobile phases consisted of (A) 100 mM ammonium acetate buffer (pH 4.5 adjusted with glacial acetic acid) and (B) acetonitrile. The flow rate was maintained at 0.8 ml/min with a slow gradient elution: 0% B until 3 min, 20% B at 15 min, 20% B at 17 min, 50% B at 18 min, maintained at 50% B until 22 min, 0% B at 23 min, and 6 min for column re-equilibration. With this same protocol standards of cysteine were prepared and run in yeast matrix to prepare the calibration curve.

### LC-MS/MS measurements of (mixed) disulfides

Glutathione was added in a 2.5-fold molar excess to pantetheine. In addition, the two compounds were added individually in separate samples. The mixtures were either directly frozen at −80 °C for later analysis (no pre-oxidation), or left to oxidize to form disulfides for 72 h at 30 °C. LC-MS/MS analyses were performed with a Shimadzu Nexera X2 HPLC system with binary LC20ADXR pumps, interfaced to a Q Exactive Plus hybrid quadrupole-orbitrap mass spectrometer (Thermo Scientific). A 50 × 2.1 mm HSS-T3 reversed-phase column with 1.8 μM particles (Waters) was used for separation. The column and autosampler temperatures were set at 40 °C and 10 °C, respectively. The injection volume was 2 μl, and the flow was set at 0.30 ml/min. Mobile phase A was water with 0.1% formic acid, and mobile phase B was acetonitrile with 0.1% formic acid. A gradient was used to separate the analytes in 8 min. MS and MS/MS analyses were performed with electrospray ionization in positive mode at a spray voltage of 3.5 kV, and sheath and auxiliary gas flow set at 60 and 14, respectively. The ion transfer tube temperature was 270 °C. Spectra were acquired in data-dependent mode with a survey scan at m/z 200 to 2000 at a resolution of 70,000, followed by MS/MS fragmentation of the top 5 precursor ions at a resolution of 17,500 in profile mode. A normalized collision energy (NCE) of 30 was used for fragmentation, and fragmented precursor ions were dynamically excluded for 6 s.

### RNA isolation, RNA-sequencing, and bioinformatics

Total RNA was isolated using the RNeasy MicroKit (QIAGEN) following the manufacturer’s instructions. The RNA-seq libraries were prepared at the ERIBA Research Sequencing Facility (Groningen, the Netherlands) according to the Smart-3SEQ protocol (53) using 15 ng of total RNA per sample as input. The libraries were pooled together and sequenced on an Illumina NovaSeq X Plus PE150 platform at Novogene. The sequencing reads were aligned against the *S. cerevisiae* reference genome (S288C version R64-1-1). Sequencing data can be found with the GEO accession number GSE290427. Differential expression analysis was performed with the edgeR package v.4.2.2. Lowly expressed genes were filtered out using *filterByExpr* function. The full set of libraries was normalized by the TMM normalization method, and between-group comparisons were done using pairwise contrasts and quasi-likelihood F (QLF) tests. Benjamini-Hochberg procedure was employed for multiple testing correction. Expression values were exported for further visualization as counts per million, adjusted for library scaling factors. Genes with an FC > 1.5 and an FDR <0.01 were subsequently used for further GO analysis using DAVID (54, 55).

## Data availability

The raw data that support the findings of this study are available on request to the corresponding author (H.S.) RNA sequencing data can be obtained from GEO accession: GSE290427. Reagents and materials associated with this study are available upon request to the corresponding author (H. S.).

## Supporting information

This article contains supporting information.

## Conflict of interest

The authors declare that they do not have any conflicts of interest with the content of this article.

## References

[1] Leonardi R., Zhang Y.M., Rock C.O., Jackowski S. (2005). Coenzyme A: back in action. Prog. Lipid Res..

[2] Strauss E. (2010). Coenzyme A biosynthesis and enzymology. Compr. Nat. Prod. Chem. Biol..

[3] Lipmann F., Kaplan N.O. (1946). A common factor in the enzymatic acetylation of sulfanilamide and of choline. J. Biol. Chem..

[4] Yu Y., Moretti I.F., Grzeschik N.A., Sibon O.C.M., Schepers H. (2021). Coenzyme A levels influence protein acetylation, CoAlation and 4′-phosphopantetheinylation: expanding the impact of a metabolic nexus molecule. Biochim. Biophys. Acta (BBA).

[5] Zhou B., Westaway S.K., Levinson B., Johnson M.A., Gitschier J., Hayflick S.J. (2001). A novel pantothenate kinase gene (PANK2) is defective in Hallervorden-Spatz syndrome. Nat. Genet..

[6] Iuso A., Wiersma M., Schüller H.-J., Pode-Shakked B., Marek-Yagel D., Grigat M. (2018). Mutations in PPCS, encoding phosphopantothenoylcysteine synthetase, cause autosomal-recessive dilated cardiomyopathy. Am. J. Hum. Genet..

[7] Bravo-Alonso I., Morin M., Arribas-Carreira L., Álvarez M., Pedrón-Giner C., Soletto L. (2023). Pathogenic variants of the coenzyme A biosynthesis-associated enzyme phosphopantothenoylcysteine decarboxylase cause autosomal-recessive dilated cardiomyopathy. J. Inherit. Metab. Dis..

[8] Dusi S., Valletta L., Haack T.B., Tsuchiya Y., Venco P., Pasqualato S. (2014). Exome sequence reveals mutations in CoA synthase as a cause of neurodegeneration with brain iron accumulation. Am. J. Hum. Genet..

[9] Hayflick S.J., Jeong S.Y., Sibon O.C.M. (2022). PKAN pathogenesis and treatment. Mol. Genet. Metab..

[10] Craig J.A., Snell E.E. (1951). The comparative activities of pantethine, pantothenic acid, and coenzyme A for various microorganisms. J. Bacteriol..

[11] Durr I.F., Cortas N. (1964). The reduction of pantethine by an extract of camel intestine. Biochem. J..

[12] De Villiers M., Barnard L., Koekemoer L., Snoep J.L., Strauss E. (2014). Variation in pantothenate kinase type determines the pantothenamide mode of action and impacts on coenzyme A salvage biosynthesis. FEBS J..

[13] Yu Y., van der Zwaag M., Wedman J.J., Permentier H., Plomp N., Jia X. (2022). Coenzyme A precursors flow from mother to zygote and from microbiome to host. Mol. Cell.

[14] Balibar C.J., Hollis-Symynkywicz M.F., Tao J. (2011). Pantethine rescues phosphopantothenoylcysteine synthetase and phosphopantothenoylcysteine decarboxylase deficiency in Escherichia coli but not in Pseudomonas aeruginosa. J. Bacteriol..

[15] Goetz V., Lefort B., Barth M., Gueguen N., Bris C., Blanchard E. (2025). Pantethine therapy dramatically rescues end-stage failing heart in a patient with deficiency of coenzyme A biosynthesis. ESC Heart Fail..

[16] Jeong S.Y., Hogarth P., Placzek A., Gregory A.M., Fox R., Zhen D. (2019). 4’-Phosphopantetheine corrects CoA, iron, and dopamine metabolic defects in mammalian models of PKAN. EMBO Mol. Med..

[17] Brown G.M., Snell E.E. (1951). Nature of multiple forms of the Lactobacillus bulgaricus factor (LBF). Proc. Soc. Exp. Biol. Med. U. S. A..

[18] White W.H., Gunyuzlu P.L., Toyn J.H. (2001). Saccharomyces cerevisiae Is Capable of de Novo Pantothenic Acid Biosynthesis Involving a Novel Pathway of β-Alanine Production from Spermine. J. Biol. Chem..

[19] Mannervik B., Nise G. (1969). Synthesis and some reactions of the pantetheine-glutathione mixed disulfide. Arch. Biochem. Biophys..

[20] Bourbouloux A., Shahi P., Chakladar A., Delrot S., Bachhawat A.K. (2000). Hgt1p, a high affinity glutathione transporter from the yeast Saccharomyces cerevisiae. J. Biol. Chem..

[21] Zulkifli M., Yadav S., Thakur A., Singla S., Sharma M., Bachhawat A.K. (2016). Substrate specificity and mapping of residues critical for transport in the high-affinity glutathione transporter Hgt1p. Biochem. J..

[22] Stolz J., Sauer N. (1999). The fenpropimorph resistance gene FEN2 from Saccharomyces cerevisiae encodes a plasma membrane H+-pantothenate symporter. J. Biol. Chem..

[23] Olzhausen J., Schübbe S., Schüller H.J. (2009). Genetic analysis of coenzyme A biosynthesis in the yeast Saccharomyces cerevisiae: Identification of a conditional mutation in the pantothenate kinase gene CAB1. Curr. Genet..

[24] Janke C., Magiera M.M., Rathfelder N., Taxis C., Reber S., Maekawa H. (2004). A versatile toolbox for PCR-based tagging of yeast genes: new fluorescent proteins, more markers and promoter substitution cassettes. Yeast.

[25] Summers E.F., Letts V.A., McGraw P., Henry S.A. (1988). Saccharomyces cerevisiae cho2 mutants are deficient in phospholipid methylation and cross-pathway regulation of inositol synthesis. Genetics.

[26] Kodaki T., Yamashita S. (1989). Characterization of the methyltransferases in the yeast phosphatidylethanolamine methylation pathway by selective gene disruption. Eur. J. Biochem..

[27] Ye C., Sutter B.M., Wang Y., Kuang Z., Tu B.P. (2017). A metabolic function for phospholipid and histone methylation. Mol. Cell.

[28] Isnard A.D., Thomas D., Surdin-Kerjan Y. (1996). The study of methionine uptake in Saccharomyces cerevisiae reveals a new family of amino acid permeases. J. Mol. Biol..

[29] Dawaliby R., Trubbia C., Delporte C., Noyon C., Ruysschaert J.M., Van Antwerpen P. (2016). Phosphatidylethanolamine is a key regulator of membrane fluidity in eukaryotic cells. J. Biol. Chem..

[30] Li Z., Agellon L.B., Allen T.M., Umeda M., Jewell L., Mason A. (2006). The ratio of phosphatidylcholine to phosphatidylethanolamine influences membrane integrity and steatohepatitis. Cell Metab..

[31] Srinivasan B., Baratashvili M., Van Der Zwaag M., Kanon B., Colombelli C., Lambrechts R.A. (2015). Extracellular 4′-phosphopantetheine is a source for intracellular coenzyme A synthesis. Nat. Chem. Biol..

[32] de Kroon A.I.P.M. (2007). Metabolism of phosphatidylcholine and its implications for lipid acyl chain composition in Saccharomyces cerevisiae. Biochim. Biophys. Acta (BBA).

[33] Sadhu M.J., Moresco J.J., Zimmer A.D., Yates J.R., Rine J. (2014). Multiple inputs control sulfur-containing amino acid synthesis in Saccharomyces cerevisiae. Mol. Biol. Cell.

[34] Lee T.A., Jorgensen P., Bognar A.L., Peyraud C., Thomas D., Tyers M. (2010). Dissection of combinatorial control by the Met4 transcriptional complex. Mol. Biol. Cell.

[35] Yamagata S. (1981). Low-molecular-Weight O-acetylserine sulfhydrylase and serine sulfhydrylase of Saccharomyces cerevisiae are the same protein. J. Bacteriol..

[36] Kaur J., Bachhawat A.K. (2007). Yct1p, a novel, high-affinity, cysteine-specific transporter from the yeast Saccharomyces cerevisiae. Genetics.

[37] Preitschopf W., Lückl H., Summers E., Henry S.A., Paltauf F., Kohlwein S.D. (1993). Molecular cloning of the yeast OPI3 gene as a high copy number suppressor of the cho2 mutation. Curr. Genet..

[38] Kim H.S., Huh J., Fay J.C. (2009). Dissecting the pleiotropic consequences of a quantitative trait nucleotide. FEMS Yeast Res..

[39] Snell E.E., Brown G.M. (1953). Pantethine and related forms of the Lactobacillus bulgaricus factor (LBF). Adv. Enzymol. Relat. Areas Mol. Biol..

[40] Warneke R., Herzberg C., Klein M., Elfmann C., Dittmann J., Feussner K. (2024). Coenzyme A biosynthesis in Bacillus subtilis : discovery of a novel precursor metabolite for salvage and its uptake system. mBio.

[41] Choi J.Y., Gihaz S., Munshi M., Singh P., Vydyam P., Hamel P. (2024). Vitamin B5 metabolism is essential for vacuolar and mitochondrial functions and drug detoxification in fungi. Commun. Biol..

[42] Kumar A., John L., Alam M.M., Gupta A., Sharma G., Pillai B. (2006). Homocysteine- and cysteine-mediated growth defect is not associated with induction of oxidative stress response genes in yeast. Biochem. J..

[43] Hughes C.E., Coody T.K., Jeong M.Y., Berg J.A., Winge D.R., Hughes A.L. (2020). Cysteine toxicity drives age-related mitochondrial decline by altering iron homeostasis. Cell.

[44] Varghese A., Gusarov I., Gamallo-Lana B., Dolgonos D., Mankan Y., Shamovsky I. (2025). Unravelling cysteine-deficiency-associated rapid weight loss. Nature.

[45] Bachhawat A.K., Thakur A., Kaur J., Zulkifli M. (2013). Glutathione transporters. Biochim. Biophys. Acta.

[46] Winzeler E.A., Shoemaker D.D., Astromoff A., Liang H., Anderson K., Andre B. (1979). Functional characterization of the S. cerevisiae genome by gene deletion and parallel analysis. Science.

[47] Stuckey S., Storici F. (2013). Gene knockouts, in vivo site-directed mutagenesis and other modifications using the delitto perfetto system in Saccharomyces cerevisiae. Methods Enzymol..

[48] Sikorski R.S., Hieter P. (1989). A system of shuttle vectors and yeast host strains designed for efficient manipulation of DNA in Saccharomyces cerevisiae. Genetics.

[49] Christianson T.W., Sikorski R.S., Dante M., Shero J.H., Hieter P. (1992). Multifunctional yeast high-copy-number shuttle vectors. Gene.

[50] Ausubel F.M., Brent R., Kingston R.E., Moore D.D., Seidman J.G., Smith J.A. (2003). Current protocols in molecular Biology kevin struhl. Curr. Protoc. Mol. Biol..

[51] Knittelfelder O.L., Kohlwein S.D. (2017). Lipid extraction from yeast cells. Cold Spring Harb. Protoc..

[52] Rouser G., Fleischer S., Yamamoto A. (1970). Two dimensional then layer chromatographic separation of polar lipids and determination of phospholipids by phosphorus analysis of spots. Lipids.

[53] Foley J.W., Zhu C., Jolivet P., Zhu S.X., Lu P., Meaney M.J. (2019). Gene expression profiling of single cells from archival tissue with laser-capture microdissection and Smart-3SEQ. Genome Res..

[54] Huang D.W., Sherman B.T., Lempicki R.A. (2008). Systematic and integrative analysis of large gene lists using DAVID bioinformatics resources. Nat. Protoc..

[55] Sherman B.T., Hao M., Qiu J., Jiao X., Baseler M.W., Lane H.C. (2022). DAVID: a web server for functional enrichment analysis and functional annotation of gene lists (2021 update). Nucleic Acids Res..

