# Supplementary material for: Opt1 imports CoA precursors as glutathione mixed disulfides
Source: J Biol Chem. 2025 Jul 21;301(9):110503. doi: 10.1016/j.jbc.2025.110503 (PMC12406275; doi:10.1016/j.jbc.2025.110503)
Supplement: Supporting Information [file mmc1.pdf]

Opt1 imports CoA precursors as glutathione mixed disulphides

Jouke Jan Wedman[1], Lotte de Vries[1], Bart van Lingen[1], Marianne van der Zwaag[1,4], Rubén Gómez-Sánchez[1], Ralph Hardenberg[1], Wim Huibers[2], Hjalmar Permentier[2], Erick Strauss[3], Michael Chang[4], Fulvio Reggiori[5], Anton I. de Kroon[6], Ody C. M. Sibon[1,4*], Hein Schepers[1,4*]

Supporting information

List of material included:

Table S1 (separate Excel file)
Table S2 (separate Excel file)
Table S3
Table S4 (separate Excel file)
Figure S1
Figure S1
Figure S2
Figure S3
Figure S4
Figure S5
Figure S6
Figure S7
Figure S8
Figure S9
Figure S10
Figure S11
Figure S12
Figure S13
Figure S14
Figure S15

Table S1 | Results of Whole Genome Sequencing of *ecm31*Δ and *pan6*Δ evolved to grow on (P)PanSH.

Provided separately as an Excel file.

Table S2 | Gene ontology enrichment analysis on differentially expressed genes.

Provided separately as an Excel file.

Table S3 | Components of synthetic media.

SMD medium (500 mL):

Component	Volume/amount	Source	Identifier
D(+) - Glucose Anhydrous (40% w/v)	25 mL	Formedium	GLU03
Yeast Nitrogen Base (10×)	50 mL	See recipe	
Complete solution (20×)	25 mL	See recipe	
Amino Acid Incomplete (10×)	50 mL	See recipe	
Vitamin solution (Pan dropout) (500×)	2.5 mL	See recipe	
Milli-Q water	Fill to 500 mL		
Agar	12 g	Formedium	AGA03

Agar is only applicable in the case of agar plates (autoclave agar with water separately)
Use drop-out solution instead of complete solution, if required
Glucose can be swapped for raffinose or galactose to make SMR or SMG, respectively

Yeast Nitrogen Base (10×) (500 mL):

Component	Amount	Source	Identifier
$(NH_4)_2SO_4$	25 g	Boom	20160
H_3BO_3 (5 g/L; 10.000×)	0.5 mL	Sigma-Aldrich	B7660
CuSO4 • 5H2O (6.24 g/L; 100.000×)	0.05 mL	Sigma-Aldrich	2790.0250
KI (10 g/L; 100.000×)	0.05 mL	Sigma	P8166
$FeCl_3 \cdot 6H_2O$ (3.32 g/L ;10.000×)	0.5 mL	Sigma	236489
$MnCl_2 \cdot 4H_2O$ (6.292 g/L; 10.000×)	0.5 mL	Sigma	M3634
$Na_2MoO_4 \cdot 2H_2O$ (2.148 g/L; 10.000×)	0.5 mL	Sigma-Aldrich	M1003
$ZnSO_4 \cdot 7H_2O$ (7.1 g/L; 10.000×)	0.5 mL	Sigma-Aldrich	Z0251
KH_2PO_4	5 g	Sigma-Aldrich	1.04873.1000

$MgSO_4$	2.5 g	Sigma-Aldrich	M7506
NaCl	0.5 g	Sigma-Aldrich	1.06404.1000
$CaCl_2$	0.5 g	Merck	A115783
Milli-Q water	Fill to 500 mL		

Autoclave before use

Complete solution (20×) (500 mL):

**Component**	**Amount**	**Source**	**Identifier**
Adenine sulphate	300 mg	Formedium	DOC0230
L-histidine	400 mg	Sigma	H-9511
L-leucine	1 g	Formedium	DOC0157
L-tryptophan	1 g	Formedium	DOC0189
L-lysine	600 mg	Formedium	DOC0161
Uracil	400 mg	Sigma	U-1128
Milli-Q water	Fill to 500 mL		

Remove one or more components to create a dropout solution
Filter sterilise before use

Amino Acid Incomplete (AAI) (10×) (500 mL):

**Component**	**Amount**	**Source**	**Identifier**
L-alanine	150 mg	Formedium	DOC0105
L-arganine	100 mg	Formedium	DOC0109
L-aspartic acid	500 mg	Formedium	DOC0121
L-glutamic acid	500 mg	Sigma	G8415
L-glycine	100 mg	Sigma-Aldrich	33226
L-isoleucine	150 mg	Formedium	DOC0153
L-phenylalanine	205 mg	Formedium	DOC0173
L-proline	150 mg	Formedium	DOC0177
L-serine	2 g	Formedium	DOC0181
L-threonine	1 g	Formedium	DOC0185
L-tyrosine	150 mg	Formedium	DOC0193
L-valine	750 mg	Formedium	DOC0197

L-glutamine	200 mg	Formedium	DOC0133
L-asparagine	800 mg	Formedium	DOC0177
Milli-Q water	Fill to 500 mL		

Filter sterilise before use

Vitamin solution (Pan dropout) (200×) (500 mL) :

**Component**	**Amount**	**Source**	**Identifier**
Biotin	0.4 mg	Sigma	036K1504
Folic acid	80 mg	Thermo Scientific	216630100
Myo-inositol	400 mg	Sigma Aldrich	SLCM5290
Nicotinic acid	80 mg	Thermo Scientific	128291000
4-aminobenzoic acid	40 mg	Sigma	A9878
Pyridoxine HCl	80 mg	Thermo Scientific	150770500
Riboflavin	40 mg	Sigma Aldrich	R4500
Thiamine HCl	80 mg	Thermo Scientific	148990100
Milli-Q water	Fill to 500 mL		

Filter sterilise before use

Table S4 | Oligonucleotides used in this study.

Proided separately as an Excel file.

Fig. S1

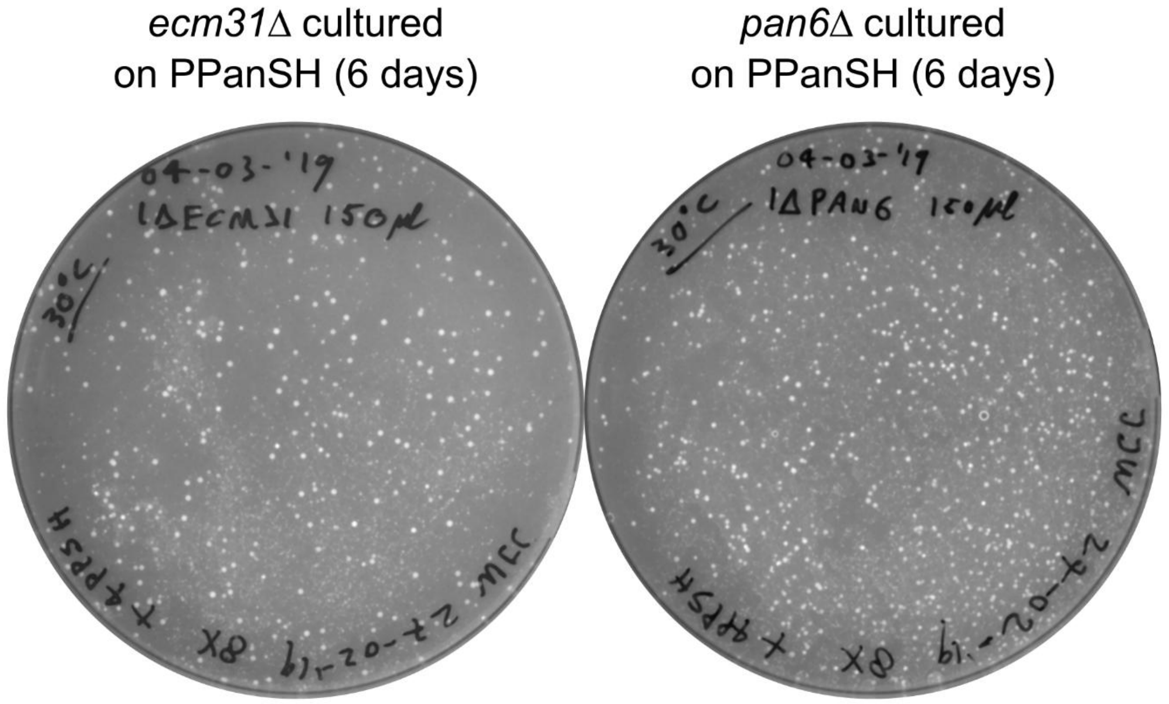

Fig. S1. Prolonged culture of *ecm31*Δ and *pan6*Δ strains on PPanSH results in the formation of suppressor mutants. On the left agar plate, *ecm31*Δ (Y03316) is cultured on pantothenate dropout plates supplemented with PPanSH. On the right agar plate, *pan6*Δ (Y02304) is cultured on pantothenate dropout plates supplemented with PPanSH. Strains were grown for 6 days at 30 °C.

Fig. S2

Glutathione disulfide (GSSG)
Chemical formula: $C_{20}H_{32}N_6O_{12}S_2$
Exact mass (M+H); 613.1592 (m/z)

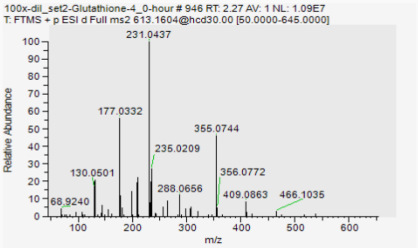

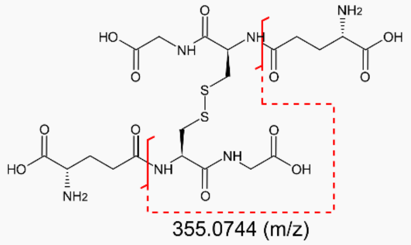

Detected (m/z)	Elemental composition	Mass error (ppm)
130.0501		
177.0332		
231.0437		
235.0209		
355.0744	$C_{10}H_{19}O_6N_4S_2$	1.07
409.0863		

Pantetheine disulfide (Pantethine; PanSSPan)
Chemical formula: $C_{22}H_{42}N_4O_8S_2$
Exact mass (M+H); 555.2517 (m/z)

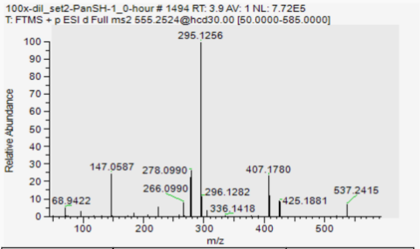

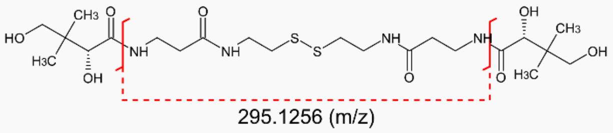

Detected (m/z)	Elemental composition	Mass error (ppm)
147.0587		
278.0990		
295.1256	$C_{10}H_{23}N_4O_2S_2$	-0.18
407.178		
425.1881		
537.2415		

Pantetheine-glutathione mixed disulfide (PanSSG)
Chemical formula: $C_{21}H_{38}N_5O_{10}S_2$
Exact mass (M+H); 584.2054 (m/z)

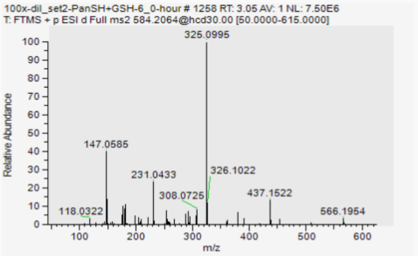

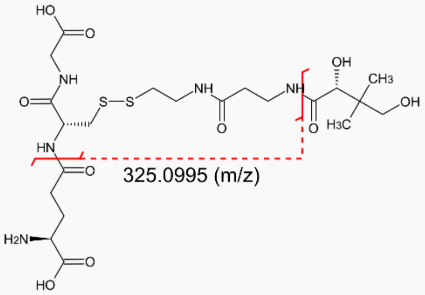

Detected (m/z)	Elemental composition	Mass error (ppm)
147.0585		
231.0433		
308.0725		
325.0995	$C_{10}H_{21}N_4O_4S_2$	-1.19
437.1522		
566.1954		

Fig. S2. LC/MS-MS of GSH and PanSH. PanSH and GSH were incubated for 72 hours alone or together to allow for disulfide formation, after which the samples were analyzed using LC-MS/MS. MS2 peaks are shown of GSSG, pantethine (PanSSPan), and the mixed disulfide PanSSG. These contain fragments with a unique m/z value matching the respective disulfides, which are displayed in the molecule structures.

Fig. S3

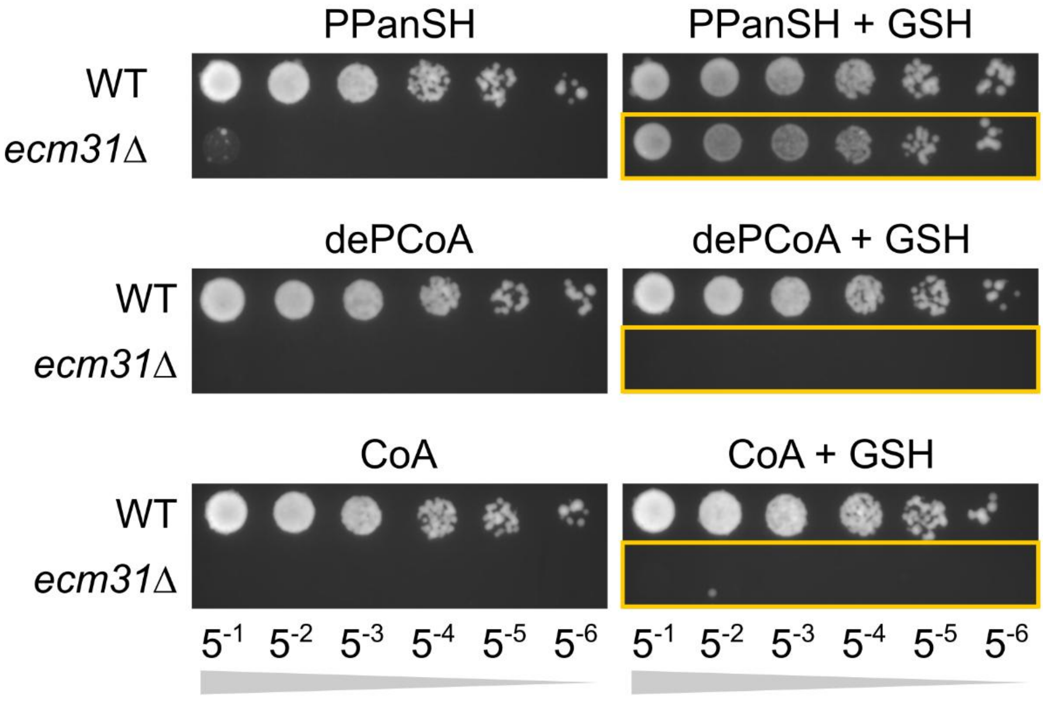

Fig. S3. Co-supplementation of GSH with PPanSH, but not dePCoA or CoA, leads to a rescue of *ecm31*Δ. Spot test of wildtype (Y00000) and *ecm31*Δ (Y03316) on pantothenate-free media supplemented with PPanSH, dePCoA, or CoA (all 27 µM), either co-supplemented with or without GSH (67 µM). Strains were grown for 8 days at 30 °C. Yellow boxes depict rescue on PPanSH when GSH is co-supplemented, which is absent in the case of depCoA or CoA.

Fig. S4

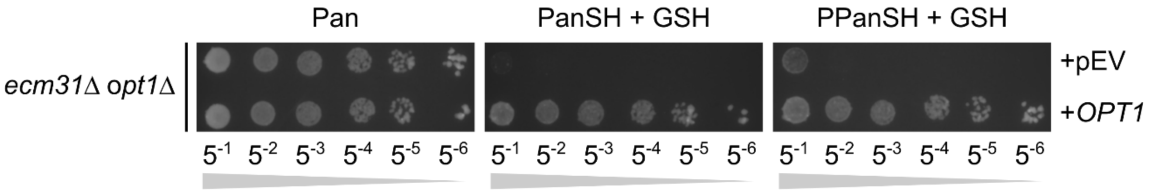

Fig. S4. *OPT1* complementation of *ecm31*Δ *opt1*Δ strain restores rescue on (P)PanSH by GSH co-supplementation. Spot test of *ecm31*Δ *opt1*Δ (JWY100) transformed with a plasmid encoding Opt1 or the empty vector control cultured on pantothenate, PanSH, or PPanSH (all 27 μM) co-supplemented with or without GSH (67 μM). Strains were grown for 8 days at 30 °C. Representative images of $n \geq 3$.

Fig. S5

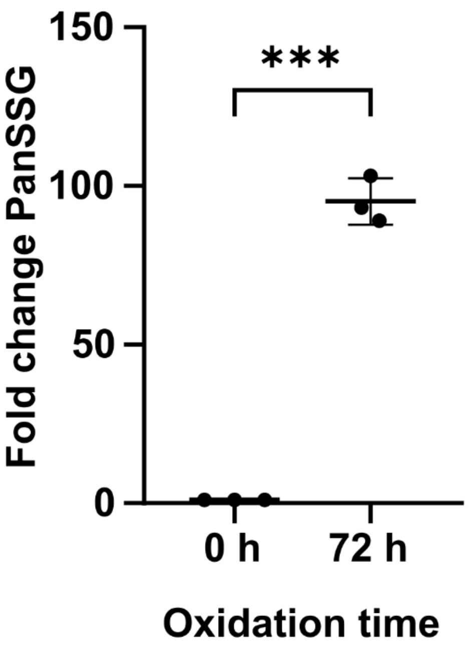

Fig. S5. Pre-oxidation of PanSH with GSH results in increased levels of the PanSSG disulfide. The fold change of PanSSG was calculated by dividing the intensity of the mass peaks and correcting for base peak intensities of GSH of both time points (0 and 72 hours of oxidation). Data is shown as mean ± SD of three biological replicates. ***$p < 0.0001$, unpaired one-tailed *t*-test was done on the intensity of the mass peaks.

Fig. S6

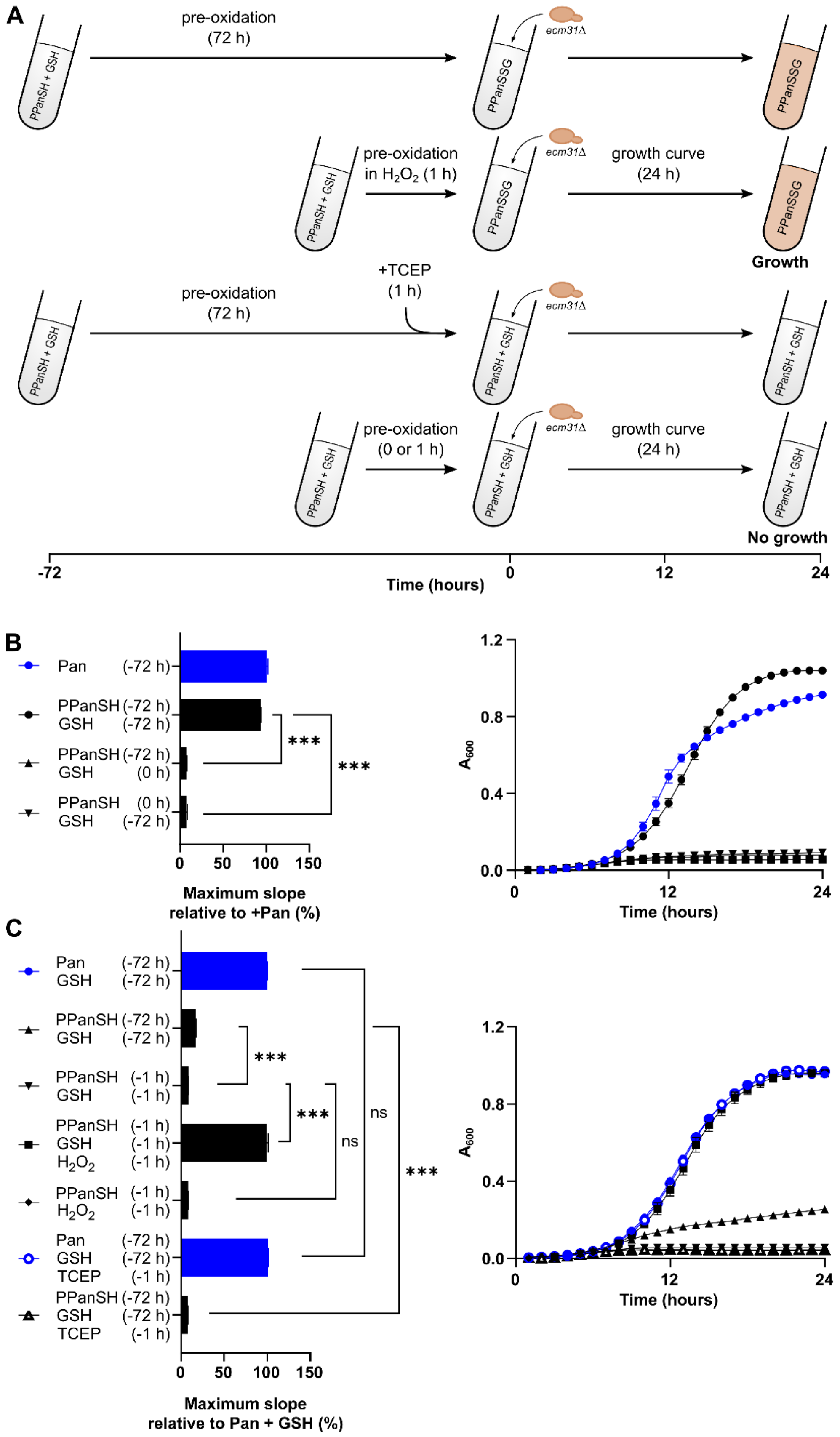

A
pre-oxidation
(72 h)
PPanSH + GSH
PPanSSG
ecm31Δ
pre-oxidation
in H_2O_2 (1 h)
growth curve
(24 h)
Growth
+TCEP
(1 h)
No growth
-72
0
12
24
Time (hours)
B
Pan (-72 h)
PPanSH (-72 h) GSH (-72 h)
PPanSH (-72 h) GSH (0 h)
PPanSH (0 h) GSH (-72 h)
***
***
Maximum slope relative to +Pan (%)
A_{600}
Time (hours)
C
Pan (-72 h) GSH (-72 h)
PPanSH (-72 h) GSH (-72 h)
PPanSH (-1 h) GSH (-1 h)
PPanSH (-1 h) GSH (-1 h) H_2O_2 (-1 h)
PPanSH (-1 h) H_2O_2 (-1 h)
Pan (-72 h) GSH (-72 h) TCEP (-1 h)
PPanSH (-72 h) GSH (-72 h) TCEP (-1 h)
ns
Maximum slope relative to Pan + GSH (%)

Fig. S6. Growth of *ecm31*Δ by PPanSH and GSH co-supplementation depends on pre-oxidized medium. *A*, the upper cartoon depicts the predicted growth of *ecm31*Δ as a result of pre-oxidation of PPanSH with GSH for 72 hours, or the addition of H_2O_2 (40 µM) for 1 hour prior to the start of a 24-hour growth curve. The lower cartoon depicts the predicted absence of growth of *ecm31*Δ when no pre-oxidation is performed, or when TCEP (100 µM) is added after pre-oxidation 1 hour prior to the start of a 24-hour growth curve. *B*, the *left* graph shows the maximum slopes of the growth curves of *ecm31*Δ (Y03316) cultured with pantothenate or PPanSH (both 27 µM) co-supplemented with GSH (67 µM), relative to pantothenate (set to 100%). The *right* graph shows corresponding growth curves. Pre-oxidation was performed directly in the media. Included are controls in which either PPanSH or GSH was pre-oxidized, with GSH and PPanSH co-supplemented at the start of the growth curve, respectively. *C*, the *left* graph shows the maximum slopes of the growth curves of *ecm31*Δ (Y03316) cultured with pantothenate or PPanSH (both 27 µM) co-supplemented with GSH (67 µM), relative to pantothenate (set to 100%). Included are conditions where pre-oxidation was shortened to 1 hour and H_2O_2 (40 µM was included, as well as conditions in which TCEP (100 µM) was added 1 hour prior to the start of the growth curve in medium pre-oxidized for 72 hours. The *right* graph shows corresponding growth curves. Pre-oxidation was performed in water. Data is shown as mean ± SD of three biological replicates. ***$p < 0.0001$, unpaired one-tailed *t*-test. TCEP; Tris(2-carboxyethyl)phosphine, H_2O_2; hydrogen peroxide. The pantothenate controls are the same as in **Fig. 3**, as this is the same experiment showing the data comparing PPanSH, not PanSH, to the control

Fig. S7

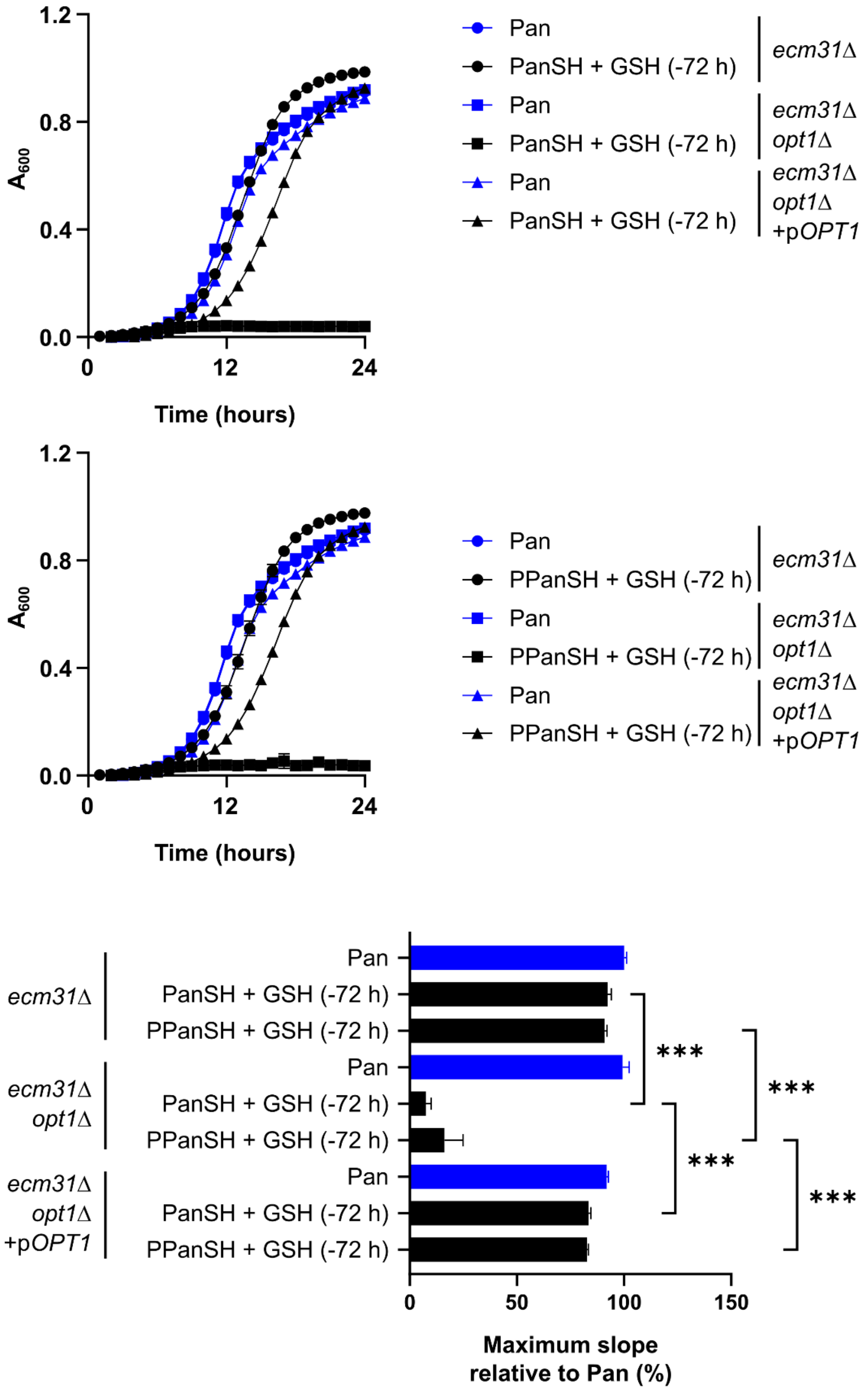

1.2
0.8
0.4
0.0
A600
0
12
24
Time (hours)
Pan
PanSH + GSH (-72 h)
Pan
PanSH + GSH (-72 h)
Pan
PanSH + GSH (-72 h)
ecm31Δ
ecm31Δ opt1Δ
ecm31Δ opt1Δ +pOPT1
1.2
0.8
0.4
0.0
A600
0
12
24
Time (hours)
Pan
PPanSH + GSH (-72 h)
Pan
PPanSH + GSH (-72 h)
Pan
PPanSH + GSH (-72 h)
ecm31Δ
ecm31Δ opt1Δ
ecm31Δ opt1Δ +pOPT1
ecm31Δ
Pan
PanSH + GSH (-72 h)
PPanSH + GSH (-72 h)
ecm31Δ opt1Δ
Pan
PanSH + GSH (-72 h)
PPanSH + GSH (-72 h)
ecm31Δ opt1Δ +pOPT1
Pan
PanSH + GSH (-72 h)
PPanSH + GSH (-72 h)
***
***
***
***
0
50
100
150
Maximum slope relative to Pan (%)

Fig. S7. *ecm31*Δ *opt1*Δ strains with *OPT1* plasmid complementation in liquid culture. Growth curves of *ecm31*Δ (Y03316) and *ecm31*Δ *opt1*Δ (JWY100) transformed with a vector encoding Opt1 or the empty vector control cultured on pantothenate (27 µM), PanSH, or PPanSH (both 27 µM) either co-supplemented and pre-oxidized with or without GSH (67 µM). Pre-oxidation was performed directly in the media. Note that the pantothenate controls in the two growth curves are the same, as this is the same experiment; the upper and lower graphs compare PanSH and PPanSH with pantothenate, respectively. The lower bar chart shows the maximum slopes of the growth curves relative to the pantothenate control (set to 100%). Data is shown as mean ± SD of three biological replicates. ***p < 0.0001, unpaired one-tailed t-test.

Fig. S8

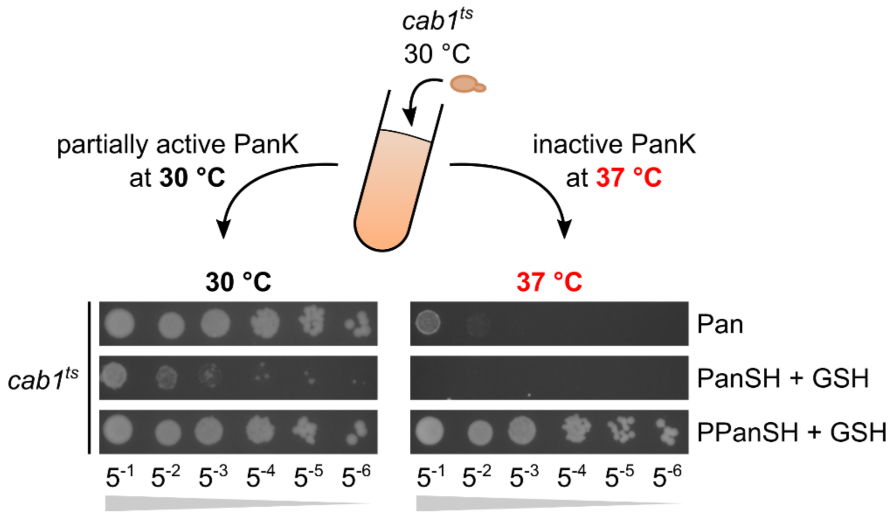

Fig. S8. A *cab1* thermosensitive strain is rescued by PPanSH with GSH co-supplementation. Spot test of a *cab1^{ts}* (G351S) strain (JS91.14-24) on pantothenate-free media supplemented with Pan, PanSH, or PPanSH (all 27 µM) co-supplemented with GSH (67 µM). Strains were grown for 4 days at 30 °C (PanK activity) or 37 °C (no PanK activity).

Fig. S9

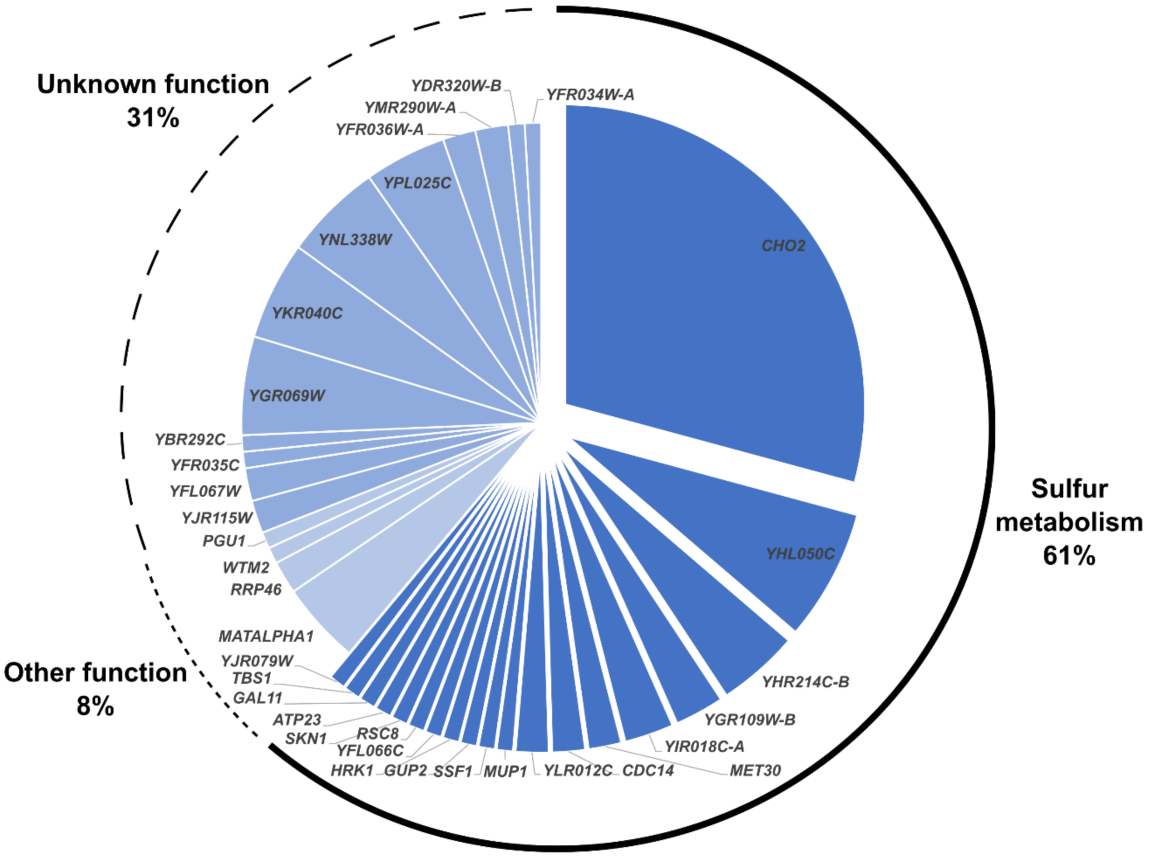

Fig. S9. Pie chart showing the mutations found in the suppressor strains of *ecm31*Δ (Y03316) and *pan6*Δ (Y02304) able to grow on (P)PanSH. See **Table S1** for raw data.

Fig. S10

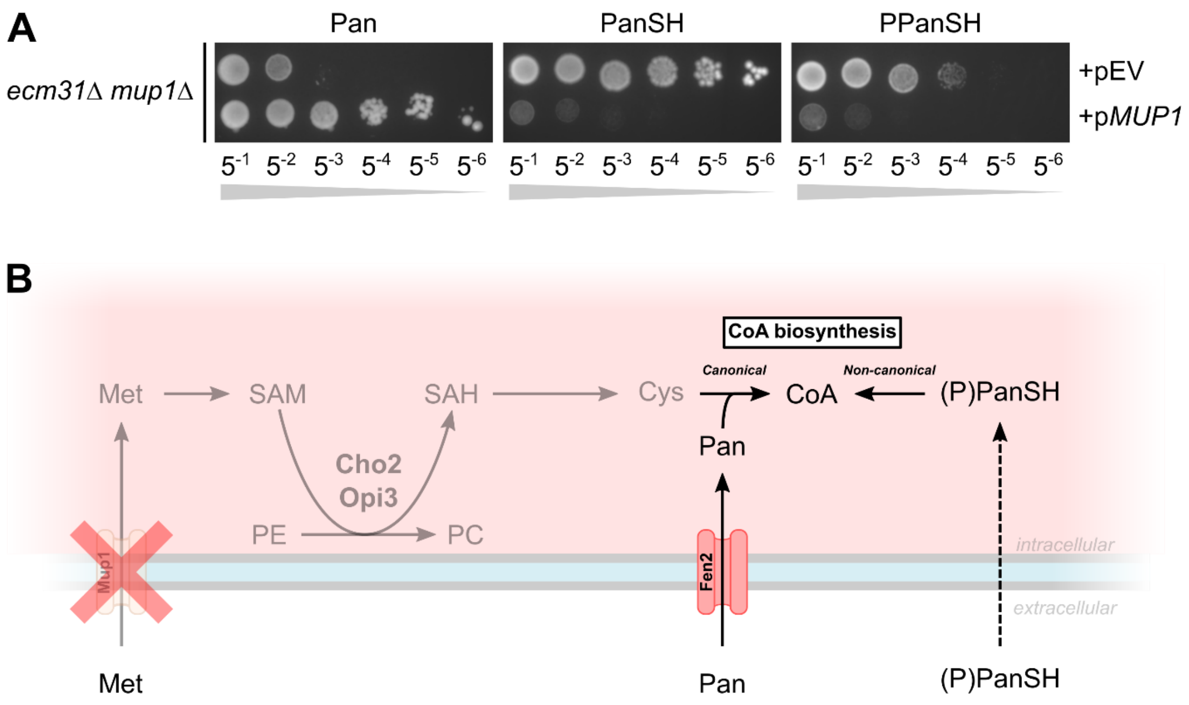

Fig. S10. *ecm31Δ mup1Δ* strain with *MUP1* plasmid complementation. *A*, spot test of *ecm31Δ mup1Δ* (JWY082) cultured on pantothenate, PanSH, or PPanSH (all 27 µM). A *MUP1* plasmid complemented strain is included. Strains were grown for 8 days at 30 °C. Representative images of n ≥ 3. *B*, cartoon depicting the uptake of methionine via Mup1. Methionine is converted into cysteine, which, together with pantothenate, is required for canonical CoA biosynthesis. Non-canonical CoA biosynthesis via uptake of (P)PanSH, however, bypasses the requirement of cysteine.

Fig. S11

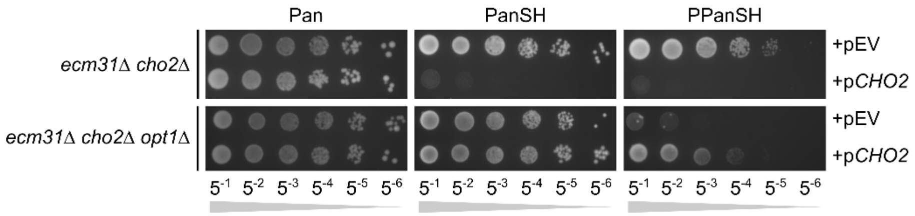

Fig. S11. *ecm31*Δ *cho2*Δ and corresponding *opt1*Δ strain with *CHO2* and *OPT1* plasmid complementation. Upper spot test is of *ecm31*Δ *cho2*Δ (JWY025), cultured on pantothenate, PanSH, or PPanSH (all 27 μM). A *CHO2* plasmid complemented strain is included. Lower spot test is of *ecm31*Δ *cho2*Δ *opt1*Δ (JWY101) cultured on pantothenate, PanSH, or PPanSH (all 27 μM). An *OPT1* plasmid complemented strain is included. Strains were grown for 8 days at 30 °C. Representative images of n ≥ 3.

Fig. S12

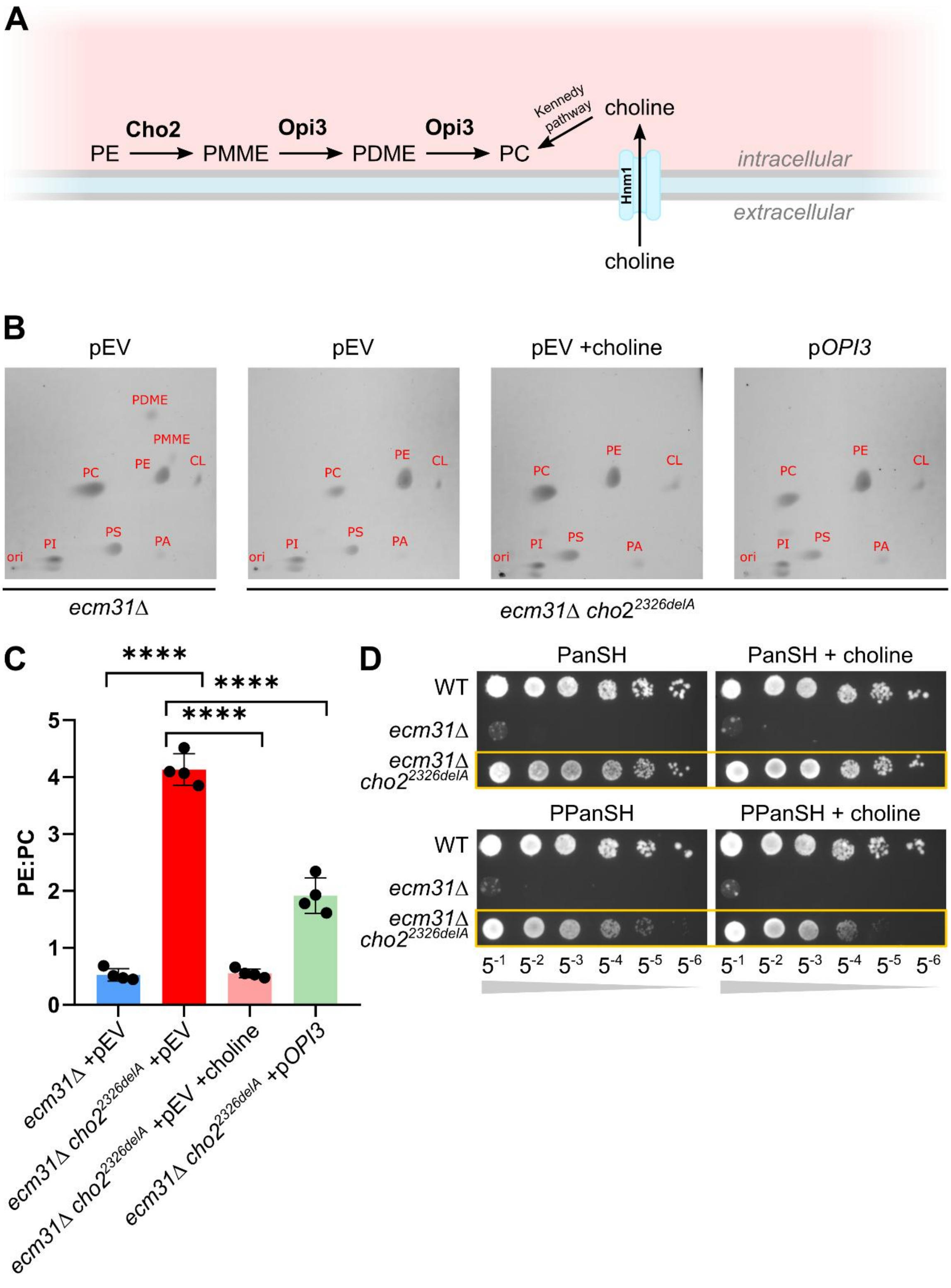
A
Cho2
Opi3
Opi3
PE
PMME
PDME
PC
Kennedy pathway
choline
Hnm1
intracellular
extracellular
choline
B
pEV
pEV
pEV +choline
pOPI3
PDME
PMME
PC
PE
CL
PS
PI
PA
ori
ecm31Δ
ecm31Δ cho2^2326delA
C
****
****
****
PE:PC
ecm31Δ +pEV
ecm31Δ cho2^2326delA +pEV
ecm31Δ cho2^2326delA +pEV +choline
ecm31Δ cho2^2326delA +pOPI3
D
PanSH
PanSH + choline
PPanSH
PPanSH + choline
WT
ecm31Δ
ecm31Δ cho2^2326delA
5^-1 5^-2 5^-3 5^-4 5^-5 5^-6

Fig. S12. The rescue of *ecm31*Δ *cho2*Δ on (P)PanSH is independent of the PE:PC. *A*, cartoon depicting the biosynthesis routes of PC. PE can be methylated by Cho2 into PMME, which is further methylated into PC by Opi3. Alternatively, choline can be used to biosynthesize PC via the Kennedy pathway. *B*, 2D-TLC analysis of total lipid extracts of *ecm31*Δ (Y03316) and *ecm31*Δ $cho2^{2326delA}$ (JWY011). Included is *ecm31*Δ $cho2^{2326delA}$ cultured with supplemented choline or with *OPI3* on a multicopy plasmid (p*OPI3*). pEV, empty vector plasmid; Ori, origin; PI, phosphatidylinositol; PS, phosphatidylserine; PA, Phosphatidic acid; CL, cardiolipin; PE, phosphatidylethanolamine; PMME, phosphatidylmonomethylethanolamine; PDME, phosphatidyldimethylethanolamine; PC, phosphatidylcholine. *C*, PE:PC determined by phosphate analysis of spots TLC from *ecm31*Δ (Y03316), *ecm31*Δ $cho2^{2326delA}$ (JWY011), and *ecm31*Δ $cho2^{2326delA}$ cultured in choline (1 mM) or carrying *OPI3* on a multicopy plasmid (p*OPI3*) (n = 4). Data is shown as mean ± SD of three biological replicates. ****p < 0.0001, unpaired one-tailed *t*-test. *D*, Spot test of wildtype (Y00000), *ecm31*Δ (Y03316), and *ecm31*Δ $cho2^{2326delA}$ (JWY011) on pantothenate media supplemented with PanSH or PPanSH (both 27 μM), and either co-supplemented with or without choline chloride (1 mM). Strains were grown for 8 days at 30 °C. Representative images of n ≥ 3.

Figure S13

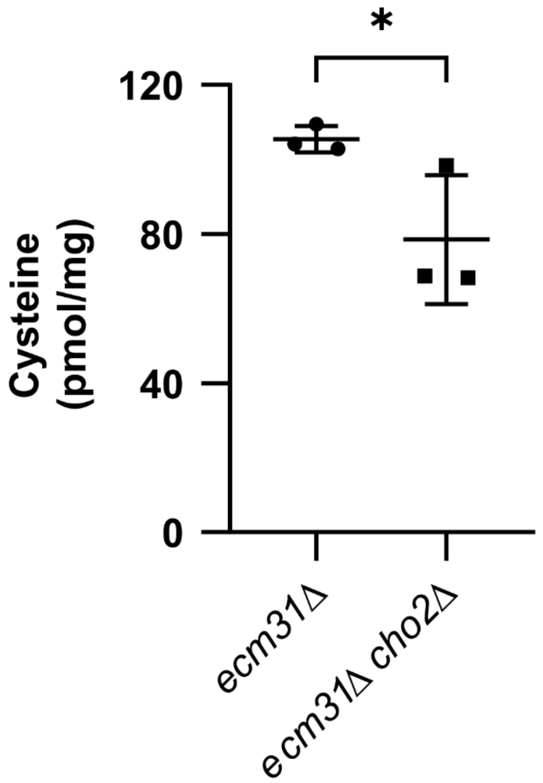

Fig. S13. ***CHO2*** **deletion leads to decreased cysteine levels.** Cysteine concentrations of *ecm31*Δ (Y03316) and *ecm31*Δ *cho2*Δ (JWY025) were measured by LCMS and compared. Data is shown as mean ± SD of three biological replicates. *$p < 0.05$, unpaired one-tailed *t*-test.

Fig. S14

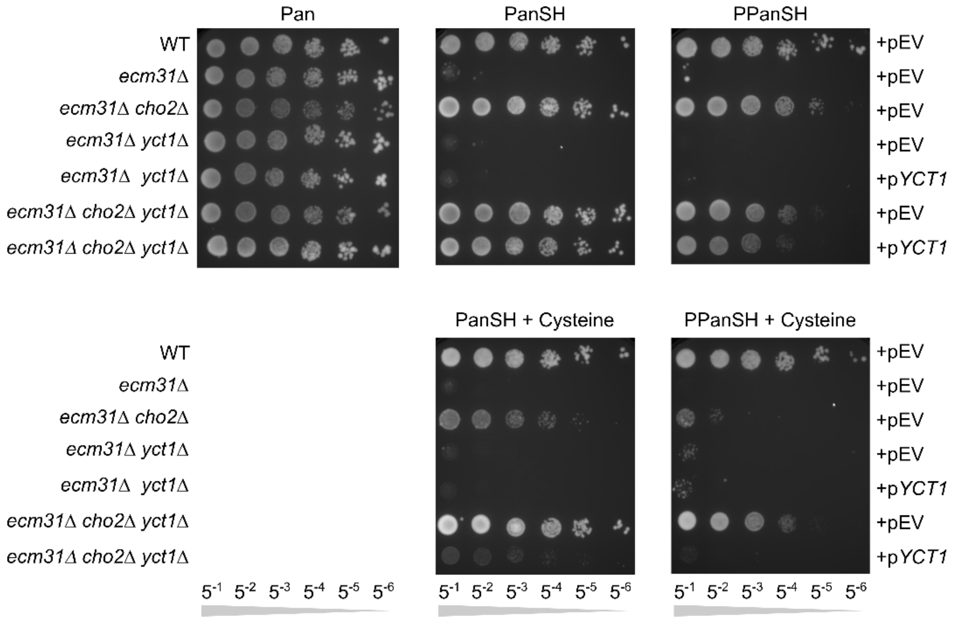

Fig. S14. *yct1*Δ strains with *YCT1* plasmid complementation. Spot test of wildtype (Y00000), *ecm31*Δ (Y03316), *ecm31*Δ *cho2*Δ (JWY025), *ecm31*Δ *yct1*Δ (JWY088), and *ecm31*Δ *cho2*Δ *yct1*Δ (JWY089) cultured on pantothenate, PanSH, or PPanSH (all 27 μM) co-supplemented with or without cysteine (67 μM). *yct1*Δ strains with *YCT1* plasmid complementation are included. Strains were grown for 8 days at 30 °C. Representative images of n ≥ 3.

Fig. S15

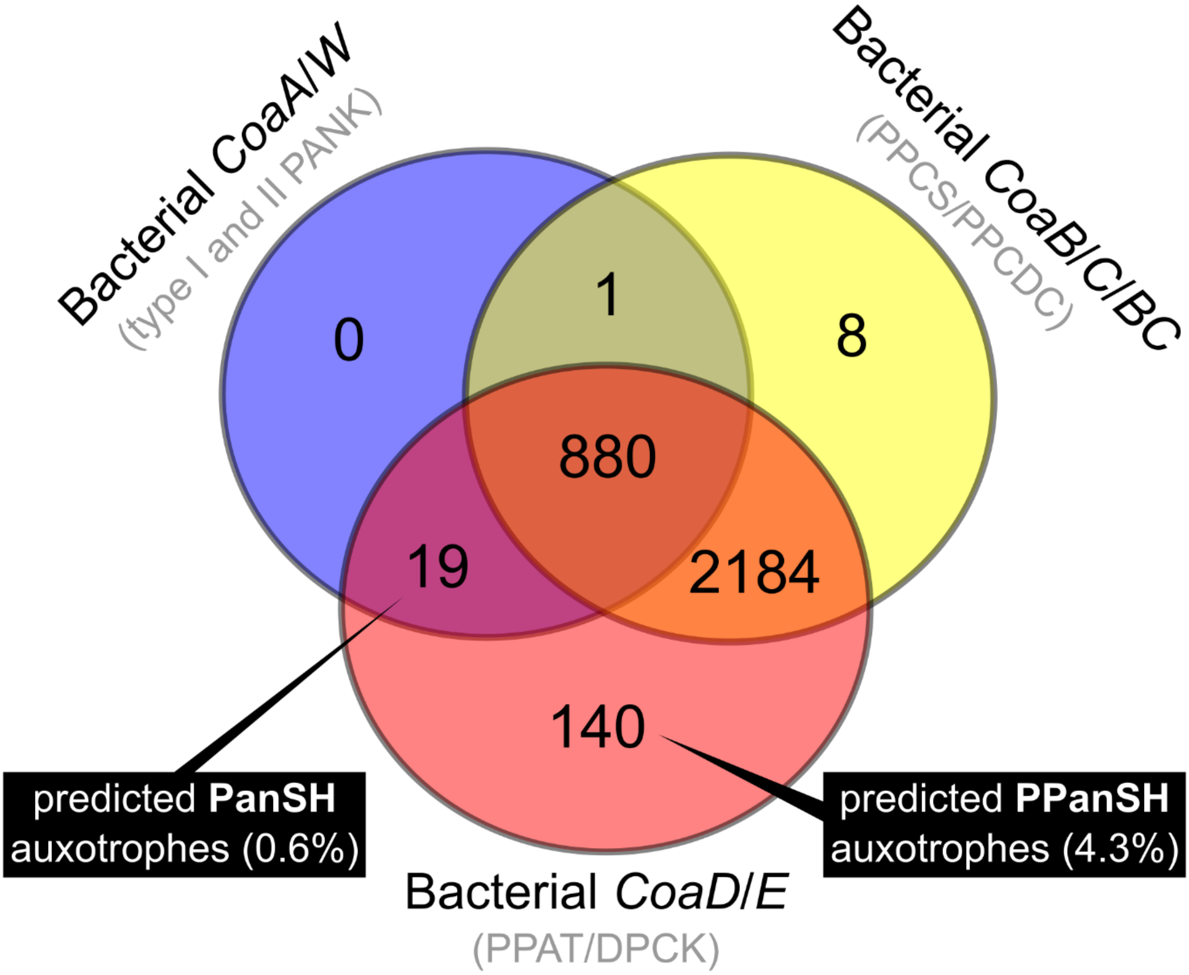

Fig. S15. Venn diagram showing shared CoA-biosynthesis genes among bacterial genomes. The percentage of bacteria predicted to be auxotrophic for (P)PanSH is shown below. Only type I and II Pank (CoaA and CoaW) are included since type III Pank (CoaX) is not able to phosphorylate pantetheine.
